# Synthesis and Evaluation of the 2-Aminothiazoles as Anti-Tubercular Agents

**DOI:** 10.1371/journal.pone.0155209

**Published:** 2016-05-12

**Authors:** Edward A. Kesicki, Mai A. Bailey, Yulia Ovechkina, Julie V. Early, Torey Alling, Julie Bowman, Edison S. Zuniga, Suryakanta Dalai, Naresh Kumar, Thierry Masquelin, Philip A. Hipskind, Joshua O. Odingo, Tanya Parish

**Affiliations:** 1 TB Discovery Research, Infectious Disease Research Institute, Seattle, Washington, United States of America; 2 Jubilant Chemsys Limited, B-34, Sector 58, Noida, India; 3 Lilly Research Laboratories, Eli Lilly and Company, Indianapolis, Indiana, United States of America; Purdue University, UNITED STATES

## Abstract

The 2-aminothiazole series has anti-bacterial activity against the important global pathogen *Mycobacterium tuberculosis*. We explored the nature of the activity by designing and synthesizing a large number of analogs and testing these for activity against *M*. *tuberculosis*, as well as eukaryotic cells. We determined that the C-2 position of the thiazole can accommodate a range of lipophilic substitutions, while both the C-4 position and the thiazole core are sensitive to change. The series has good activity against *M*. *tuberculosis* growth with sub-micromolar minimum inhibitory concentrations being achieved. A representative analog was selective for mycobacterial species over other bacteria and was rapidly bactericidal against replicating *M*. *tuberculosis*. The mode of action does not appear to involve iron chelation. We conclude that this series has potential for further development as novel anti-tubercular agents.

## Introduction

Tuberculosis (TB) remains a deadly global threat and continues to be one of the leading causes of death worldwide [1]. Although significant strides have been made toward combating this disease, coordinated global health efforts have fallen short, in part due to patient incompliance to current treatment regimens [1]. The current minimum treatment regimen for TB requires a combination therapy of isoniazid (INH), rifampicin (RIF), pyrazinamide (PZA), and ethambutol (EMB) for two months, followed by INH and RIF for an additional four months [2]. This intensive therapy is becoming increasingly ineffective with the emergence of both multidrug-resistant TB (MDR-TB) and extensively drug-resistant TB (XDR-TB). MDR-TB is resistant to isoniazid and rifampicin, whereas XDR-TB is resistant to isoniazid, rifampicin, one of the fluoroquinolones, as well as one injectable drug. Both MDR-TB and XDR-TB have the potential to render our current treatment regimen ineffective. Therefore, the discovery and development of novel TB drugs is urgent.

Researchers have relied on a variety of screening techniques to identify promising compound series for TB drug development. A large number of compound series have been identified via high-throughput phenotypic screening assays [3–5]. Among the compound classes identified from such phenotypic screens, the aminothiazole (AT) series is promising for its activity [5]. The AT core is a common motif currently found in several FDA-approved drugs, such as the nonsteroidal anti-inflammatory drug meloxicam [6], the tyrosine kinase inhibitor dasatinib [7], and cefdinir, a 3rd-generation cephalosporin [8,9]. Most recently, AT derivatives with activity against *M*. *tuberculosis* were described [10].

One of the key features of the AT motif is its versatility to accommodate a large number of different functional groups without significant loss of activity [10,11]. In addition, the ease with which AT analogs can be synthesized is an attractive quality, as it lends itself to the rapid exploration of structure-activity relationship (SAR) studies. Furthermore, AT analogs can be achieved in a few steps from inexpensive, commercially available starting materials, which may be well suited for large scale production. We focused our efforts on a full evaluation of this series to identify compounds with good activity against *M*. *tuberculosis* and good selectivity over mammalian cells.

## Results & Discussion

### Chemistry

We initiated our SAR investigations by synthesis of reference compound **1,** which was selected based on the literature report of good activity against *M*. *tuberculosis* (MIC < 0.1 μM) [5]. We confirmed the activity, albeit with a shift in our assay (MIC = 8 μM).

**Fig 1:**
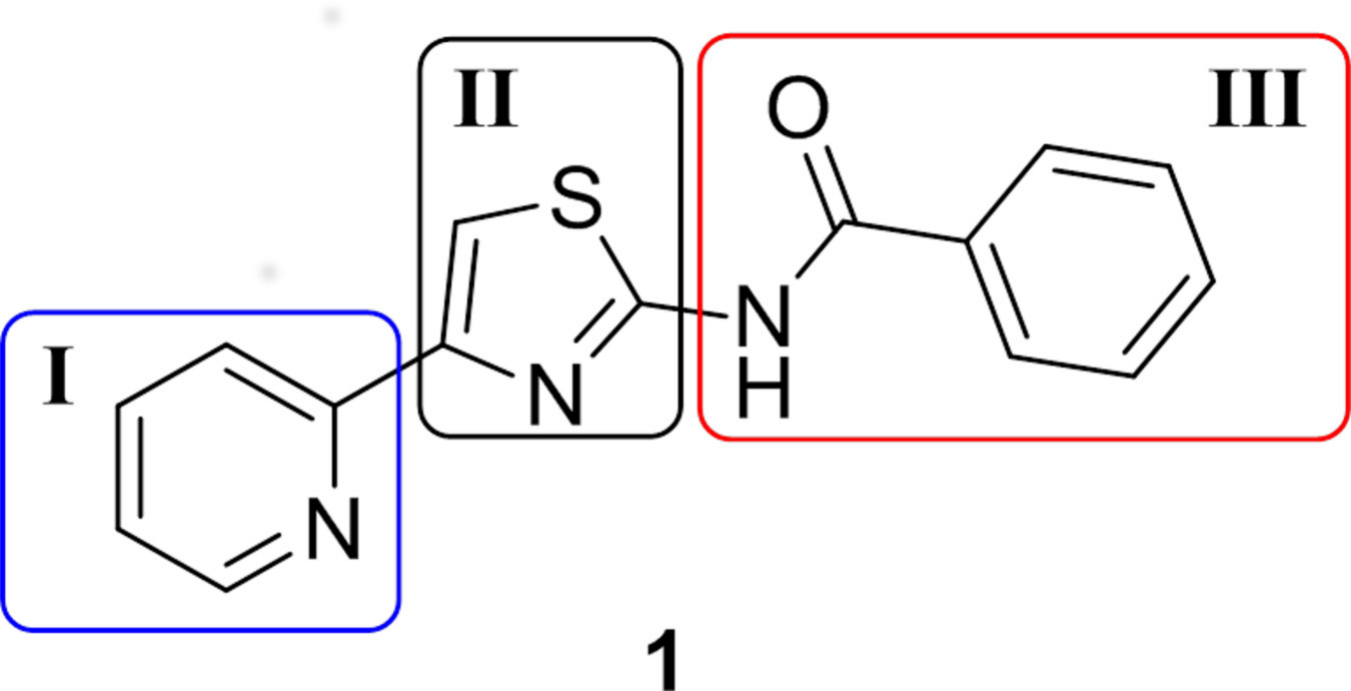
Reference compound 1. Three core segments illustrated with box and numbering.

Beginning with **1** as a confirmed active compound, we designed and synthesized analogs to explore the chemical space, and establish the structural and functional requirement for activity against *M*. *tuberculosis*. We explored three main segments to the series, as depicted in Fig 1, namely the substitutions at the C-2 (III) and C-4 (I) positions as well as various replacements for the thiazole core (II).

**Fig 2:**
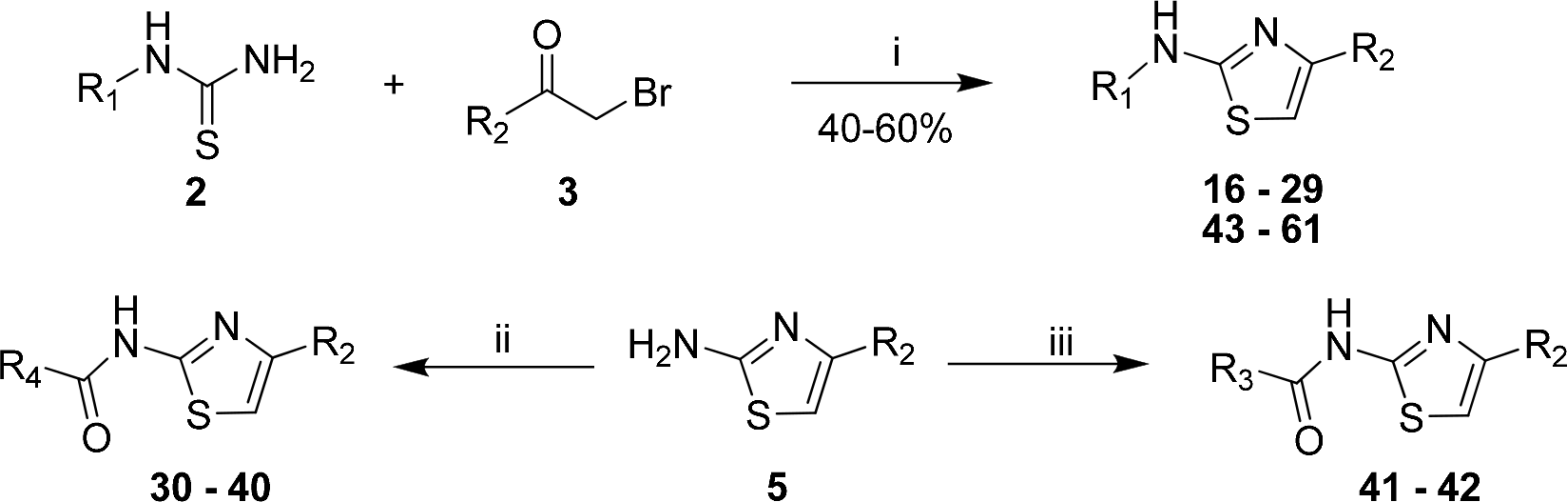
Chemical synthesis of 2-aminothiazole analogs. Reagents: (i) EtOH, 70°C, 2 h; (ii) R_4_COX, Et_3_N, THF, r.t., 1 h; (iii) R_3_NCO, Et_3_N, THF, r.t., 1 h.

We synthesized and evaluated a series of AT for SAR determination and identified a number of analogs that possess excellent activity against *M*. *tuberculosis*. The synthesis of AT is straightforward and is depicted in Fig 2.

Aminothiazoles **16–29** were achieved via a condensation reaction between a suitably substituted thiourea (**2**) and a substituted bromoketone (**3**) in a Hantzsch reaction [10,12–14]. Where necessary, the thiourea (**2**) was prepared by a reaction between a corresponding amine with benzoyl isothiocyanate followed by base hydrolysis of the resulting *N*-benzoylthiourea to give **2**. The amine intermediate (**5**) prepared in this manner was subsequently acylated to give **30**–**40** or treated with cyclopentyl isocyanate or phenyl isocyanate to give urea **41** or **42**, respectively. Analogs **43–61**, which examine different substituents at the C-2 position of the thiazole core, were prepared in a similar manner.

**Fig 3:**
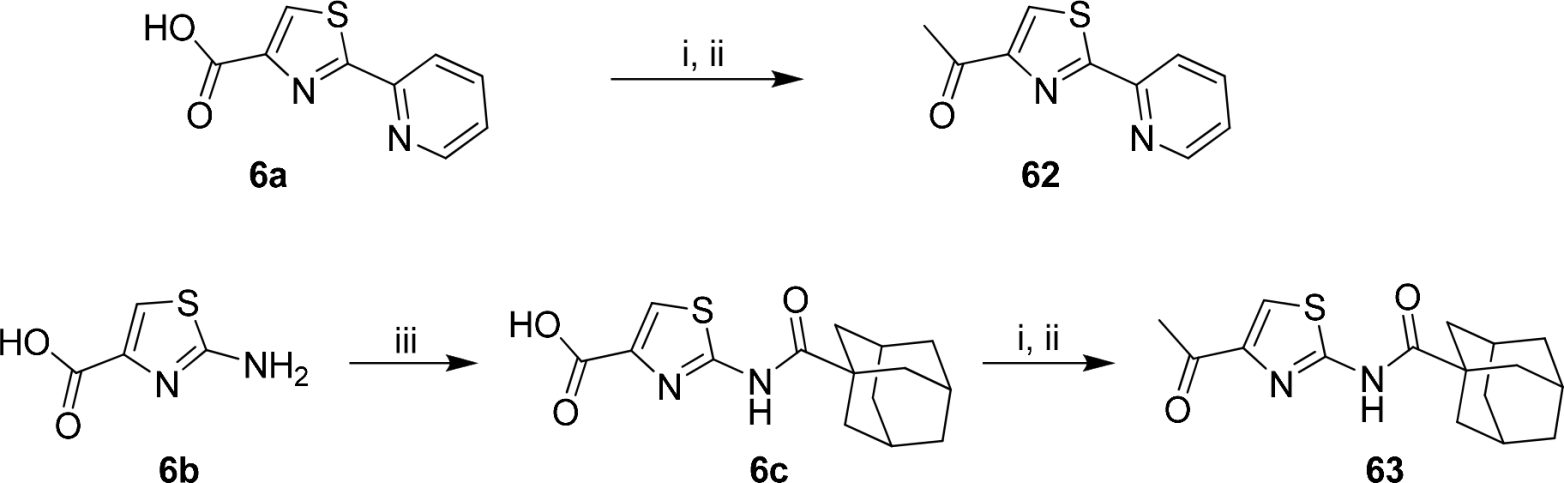
Chemical synthesis of C-4 ketone and carboxamide analogs. Reagents: (i) EDC.HCl, HOBt, NCH_3_(OCH_3_), DIPEA, CH_2_Cl_2_, 16 h; (ii) CH_3_MgBr, THF, -78°C–r.t., 2 h; (iii) 1-adamantanoyl chloride, Et_3_N, THF, r.t., 1 h.

The C-4 ketone (**62**) and carboxamide (**63**) were prepared from carboxylic acid **6**, which is accessible from commercially available ethyl 2-aminothiazole-4-carboxylate (Fig 3). Treatment of compound **6** with 2-chloropyridine in THF under reflux conditions followed by conversion to Weinreb amide and Grignard in the presence of methyl magnesium bromide gave **62** in 49% yield. Similarly, acylation of **6** gave intermediate **7**, which after a similar Weinreb amide conversion afforded **63**.

**Fig 4:**
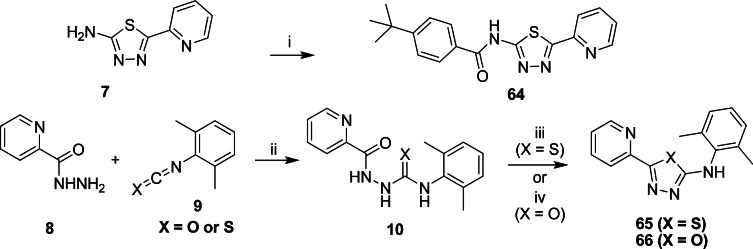
Chemical synthesis of analogs comprised of thiazole core replacement. Reagents:: (i) 4-(t-butyl)PhCOCl, EtOH, 1 h; (ii) EtOH, reflux, 16 h; (iii) H_2_SO_4_, r.t., 16 h; (iv) EDC, CH_2_Cl_2_, r.t., 16 h.

We also prepared analogs which are comprised of modifications to the thiazole core. Thiadiazole **64** was prepared from commercial 2-aminothiadiazole (**7**) via standard amidation chemistry. Thiadiazole **65** and oxadiazole **66** were prepared in two steps (Fig 4). Treating hydrazide **8** with isothiocyanate and /or isocyanate **9** gave intermediate **10**, which after acid-catalyzed ring-closure afforded thiadiazole **65**. Treatment of **10** with EDC gave oxadiazole **66**.

**Fig 5:**
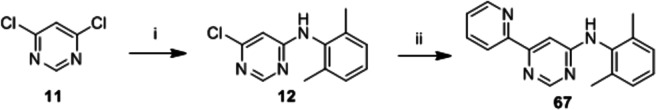
Chemical synthesis of pyrimidine-based analogs. Reagents: (i) 2,6-dimethylaniline, THF, reflux, 16 h; (ii) 2-(tributylstannyl)pyridine, Pd(PPh_3_), DMF, 100°C, 16 h.

The pyrimidine-based analog **67** was prepared according to Fig 5. Amination of dihalide **11** in the presence of 2,6-dimethylaniline gave intermediate **12**. Alkylation of **12** in the presence of 2-(tributylstannyl)pyridine under Stille coupling conditions in Fig 5 afforded **67**.

**Fig 6:**
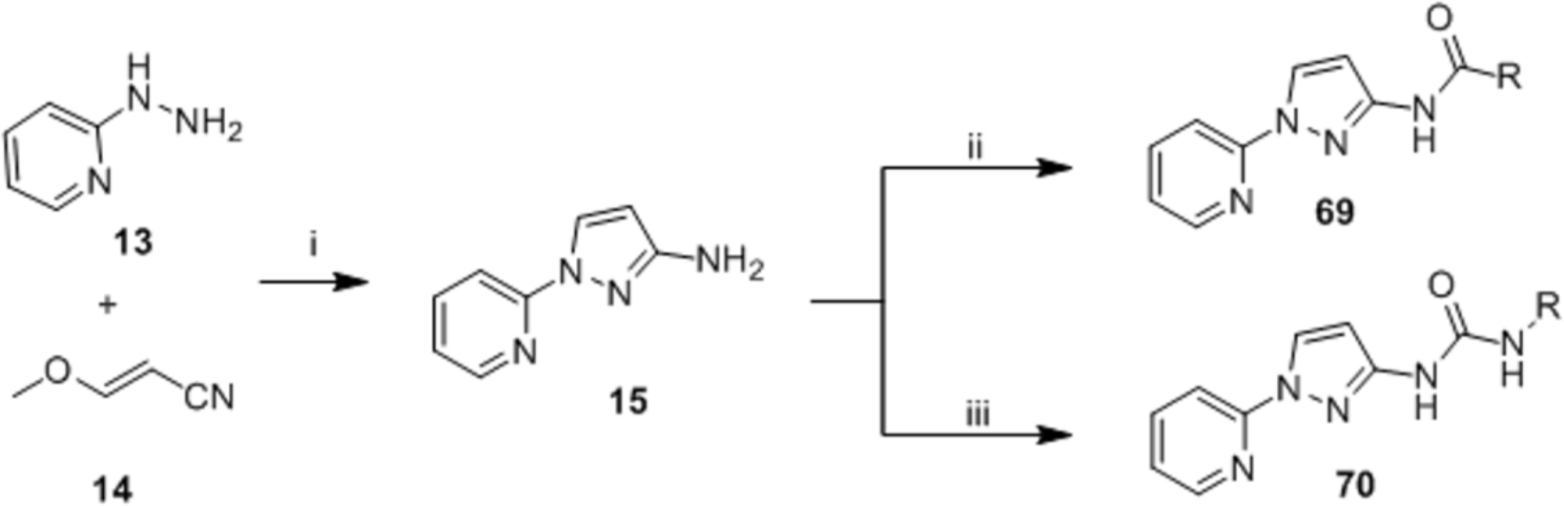
Chemical synthesis of pyrazole-based analogs. Reagents: (i) KO^*t*^Bu, BuOH, reflux, 3 h; (ii) RCOCl, THF, r.t., 1 h; (iii) RNCO, THF, 1 h.

The synthesis of pyrazole analogs **69** and **70** is shown in Fig 6. 2-Hydrazinopyridine (**13**) was reacted with methoxyacrylonitrile (**14**) in the presence of base to give pyridylpyrazole intermediate **15,** which was readily converted to **69** via standard acylation protocols. Treatment of **15** with isocyanate afforded urea **70.**

### Structure-Activity Relationship (SAR) studies

**Fig 7:**
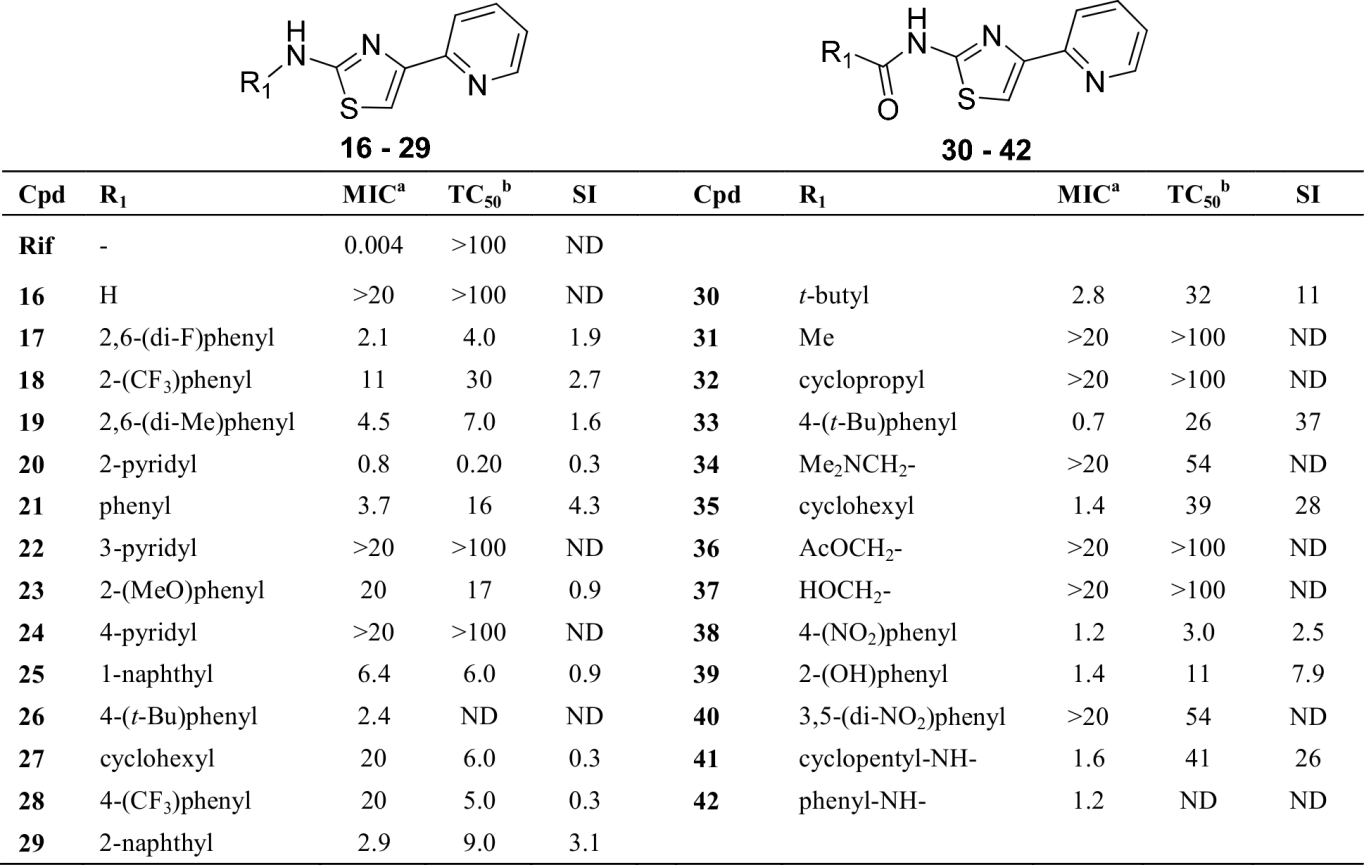
SAR at C-2 position of thiazole. ^a^Compounds were tested for activity against *M*. *tuberculosis*. Minimum inhibitory concentration (MIC, in μM) is the minimum concentration required to inhibit the growth of *M*. *tuberculosis* in liquid culture. MICs of active compounds are the average of two independent experiments. ^b^Toxic concentration (TC50, in μM) is the concentration required to inhibit growth of Vero cells by 50%. Selectivity index (SI) is the ratio of TC50 to MIC. Cpd = compound; Rif = rifampicin; ND = not determined.

The goal of our research efforts was to explore the AT compound class, and establish SAR with respect to activity against *M*. *tuberculosis* and selectivity over mammalian cells. Compounds were designed to explore various segments of the hit structure **1** including the thiazole core. The 2-pyridyl was previously reported to be a preferred residue at the C-4 position [5]. Therefore, beginning with a 2-pyridyl substituent fixed at C-4 position of the thiazole, we explored the C-2 position for optimal substitution pattern (Fig 7).

We synthesized analogs with various substitutions at the C-2 of the thiazole, including a diversity of amines (**16–29**), amides (**30–40**) and ureas (**41** and **42**). The un-substituted amine (**16)** and the aliphatic cyclohexylamine (**27)** were less active. Heteroaromatic amines such as **20** had the best activity but were also cytotoxic. The isosteric 3- and 4-pyridyl analogs **22** and **24** showed no activity indicating a preference for the 2-isomer. Aniline **21** had moderate activity comparable to the more lipophilic analog **26**.

We noted increased anti-tubercular activity in compounds with amide or urea substituents at C-2 position, as compared to amines. For example, the cyclohexylamide **35** had 14-fold greater activity as compared to the analogous amine **27**, and the amide **33** had 3-fold greater activity over the analogous amine **26**. Both **33** and **35** had good separation from cytotoxicity, with respective selectivity index (SI = MIC/TC_50_) of 37 and 28. The potency and selectivity of these compounds highlighted the benefit of incorporating an amide linkage to the core, thus providing a good starting point for compound optimization.

Urea analogs were also more active relative to their aniline counterparts; for example the cyclopentyl urea analog **41** demonstrated good activity against *M*. *tuberculosis*, as well as selectivity (SI = 26). A phenyl urea analog **42** had 3-fold higher activity as compared to the aniline **21**. Furthermore, lipophilic amides retained activity, while attempts to introduce small alkyl (**31**, and **32**) or small polar (**34**, **36** and **37**) amides generally resulted in loss of activity.

**Fig 8:**
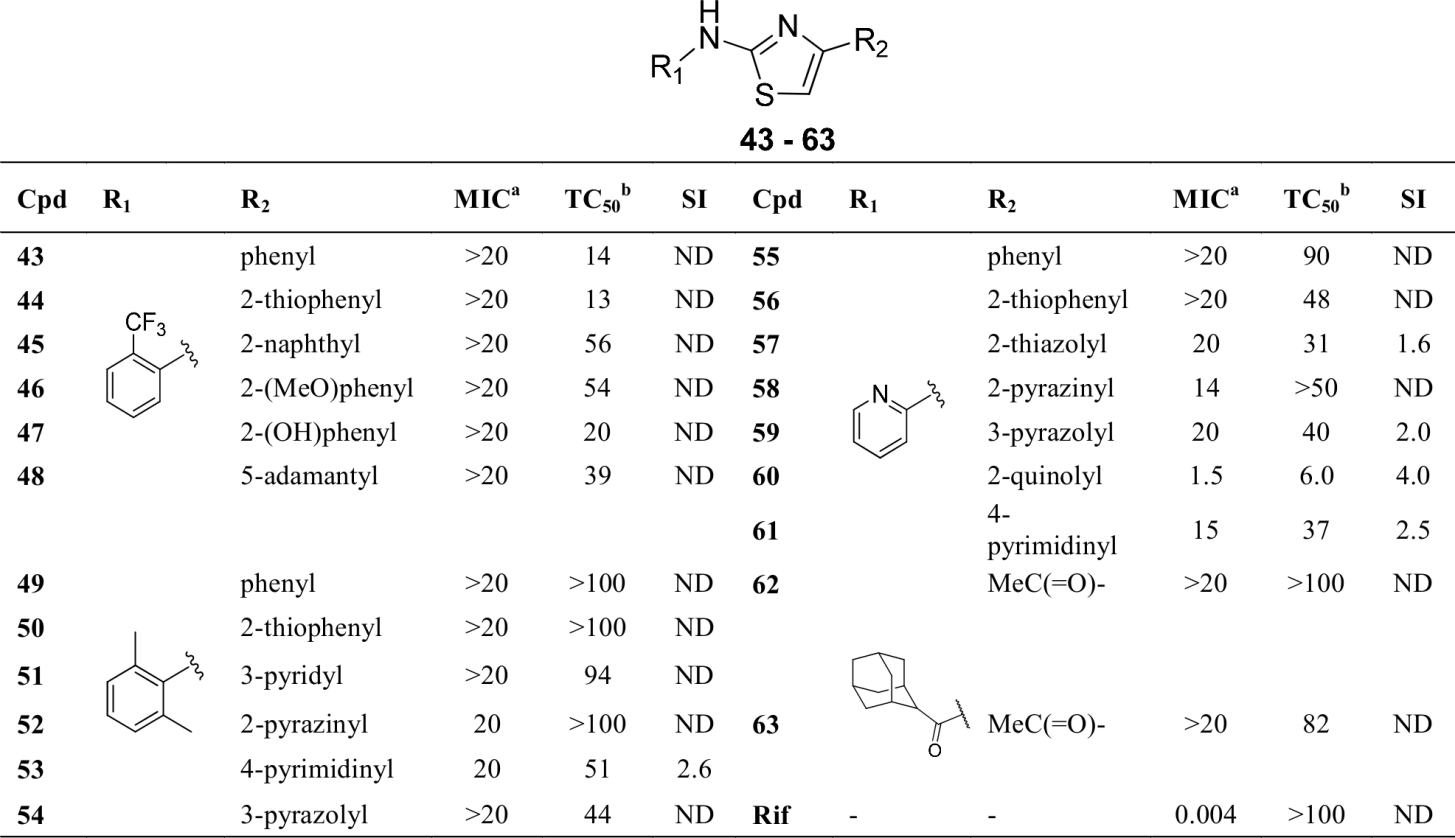
SAR at C-4 position of thiazole core. ^a^MIC (μM) is the minimum concentration required to inhibit the growth of *M*. *tuberculosis* in liquid culture. MICs of active compounds are the average of two independent experiments. ^b^Toxic concentration (TC_50_, in μM) is the concentration required to inhibit growth of Vero cells by 50%. Selectivity index (SI) is the ratio of TC_50_ to MIC. Cpd = compound; Rif = rifampicin; ND = not determined.

Consistent with prior reports, we found that the placement of a 2-pyridyl unit at C-4 position of the thiazole core conferred good activity [10,11]. Given the limited scope of these reports on C-4 SAR, we decided to further explore requirements for activity at this position. We designed, synthesized and evaluated a diversity of substituents ranging in structural and functional scope to include alkyls, aromatics, heteroaromatics and specific 2-pyridyl surrogates (Fig 8).

These analogs were designed as combinations with selected C-2 residues known to confer good activity. The 3-pyridyl (**51**) isomer was devoid of any activity, an indication of a specific preference for the 2-pyridyl residue at C-4 position. In general, C-4 alkyls, such as **48**, or plain aromatics (**43, 45, 49** and **55**) were inactive. 2-Pyridyl replacements with 2-methoxyphenyl (**46)** or 2-hydroxyphenyl (**47)** yielded analogs devoid of any activity. Several other 2-pyridyl isosteric replacements tested showed no activity. The C-4 carboxamide or ketone (**62** and **63**) analogs also tested inactive. 2-Pyrazinyl (**52, 58**), 4-pyrimidinyl (**53, 61**) and 2-quinolyl (**60**) analogs showed moderate activity. However, these compounds also showed moderate cytotoxicity. Accommodation of these residues at C-4 however provides an opportunity for modulating physicochemical properties of the compound series without much penalty in activity.

**Fig 9:**
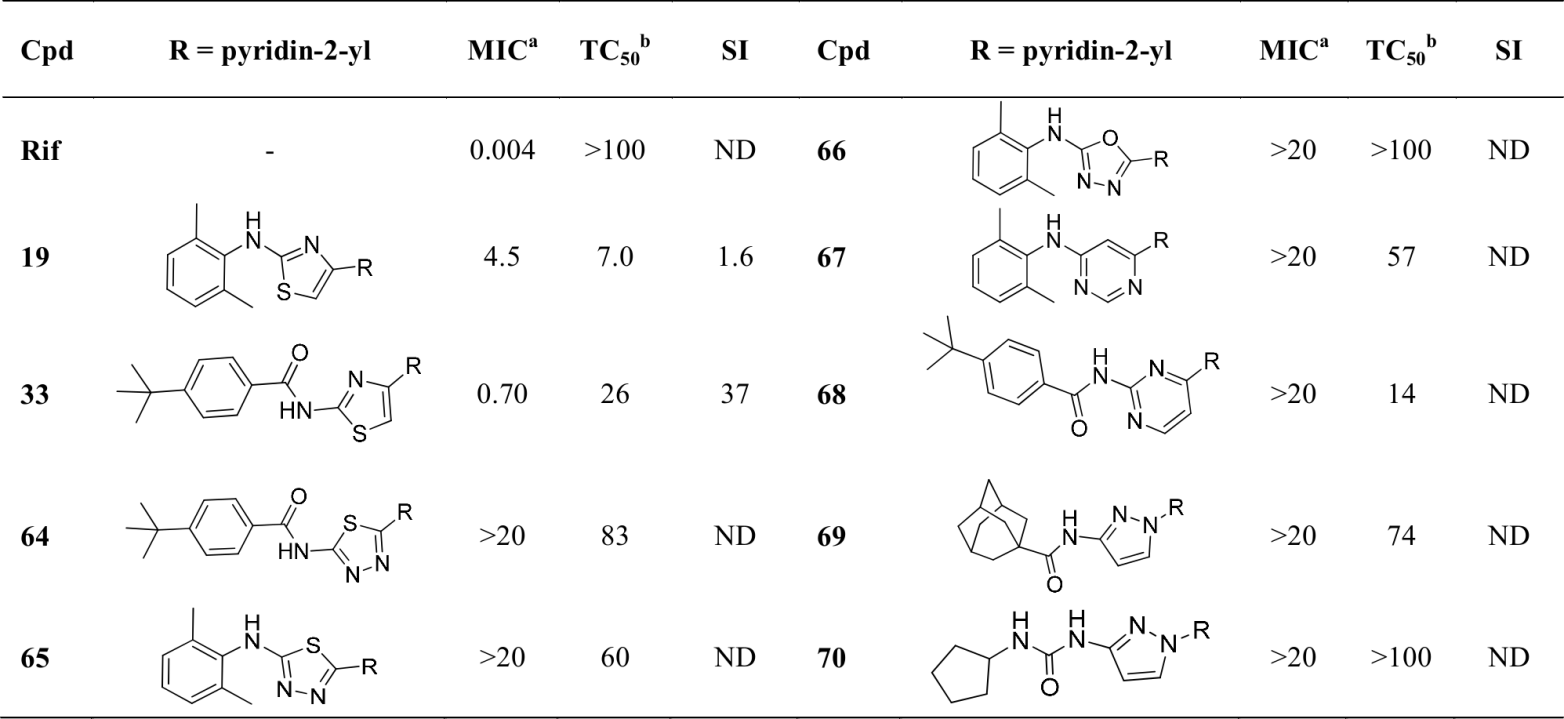
SAR of thiazole core replacements. ^a^Compounds were tested for inhibition of *M*. *tuberculosis*. Minimum inhibitory concentration (MIC, in μM) is the minimum concentration required to completely inhibit the growth of *M*. *tuberculosis* in liquid culture. MICs of active compounds are the average of two independent experiments. ^b^Toxic concentration (TC_50_, in μM) is the concentration required to inhibit growth of Vero cells by 50%. Selectivity index (SI) is the ratio of TC50 to MIC. Cpd = compound; Rif = rifampicin; ND = not determined.

We further synthesized thiazole core replacements (Fig 9). We selected optimal pairs of C-2/C-4 substitution patterns established from our SAR studies of the thiazole scaffold to investigate alternative scaffolds. Thus, 2,6-dimethylanilino, 4-tert-butylbenzamido, adamantylcarboxamido and 1-cyclopentylurea units were selected as optimized C-2 residues for pairing with 2-pyridyl unit at C-4 (Fig 9).

We investigated isosteric replacement of the thiazole scaffold in amine 1**9** (MIC = 4.5 μM) compared to thiadiazole **65**, oxadiazole **66**, and pyrimidine **67**. In addition, we investigated thiazole replacement of **33** (MIC = 0.70 μM) by comparing the activity against *M*. *tuberculosis* of related thiadiazole **64** and pyrimidine **68**. We also looked into replacing the thiazole with a pyrazole (**69** and **70**). However, all modifications resulted in total loss of activity. These findings demonstrate that isosteric replacement of the thiazole core by other five- or six-membered heterocyclics do not necessarily produce compounds with retained biological activity i.e. they are not bioisosteres. Therefore, we conclude that the thiazole core must play a significant role in the activity against *M*. *tuberculosis*, in addition to the structural display of key residues.

## Aminothiazoles have limited broad spectrum activity

Two compounds were selected for testing against other bacterial species–we used the non-pathogenic mycobacterial species *Mycobacterium smegmatis*, as well as *Staphylococcus aureus* (Gram positive) and *Escherichia coli* (Gram negative) (Table 1). Both compounds were potent against *M*. *tuberculosis*, but also had activity against *M*. *smegmatis*, suggesting that the series targets mycobacteria broadly. One compound also had activity against *S*. *aureus*, suggesting that there may be limited broad spectrum activity, but neither compound was active against *E*. *coli*.

**Table 1:** Spectrum of activity against bacterial species. The MIC against four bacterial species was determined on solid medium using the agar serial proportion method. MIC_99_ is the minimum concentration required to prevent growth 99% of bacteria.

Compound	*Mycobacterium tuberculosis* MIC_99_ (μM)	*Mycobacterium smegmatis* MIC_99_ (μM)	*Escherichia coli* MIC_99_ (μM)	*Staphylococcus aureus* MIC_99_ (μM)
**20**	1.0	2.5	>100	10
**33**	0.25	12.5	>100	>100

### Aminothiazoles have bactericidal activity against *M*. *tuberculosis*

**Fig 10:**
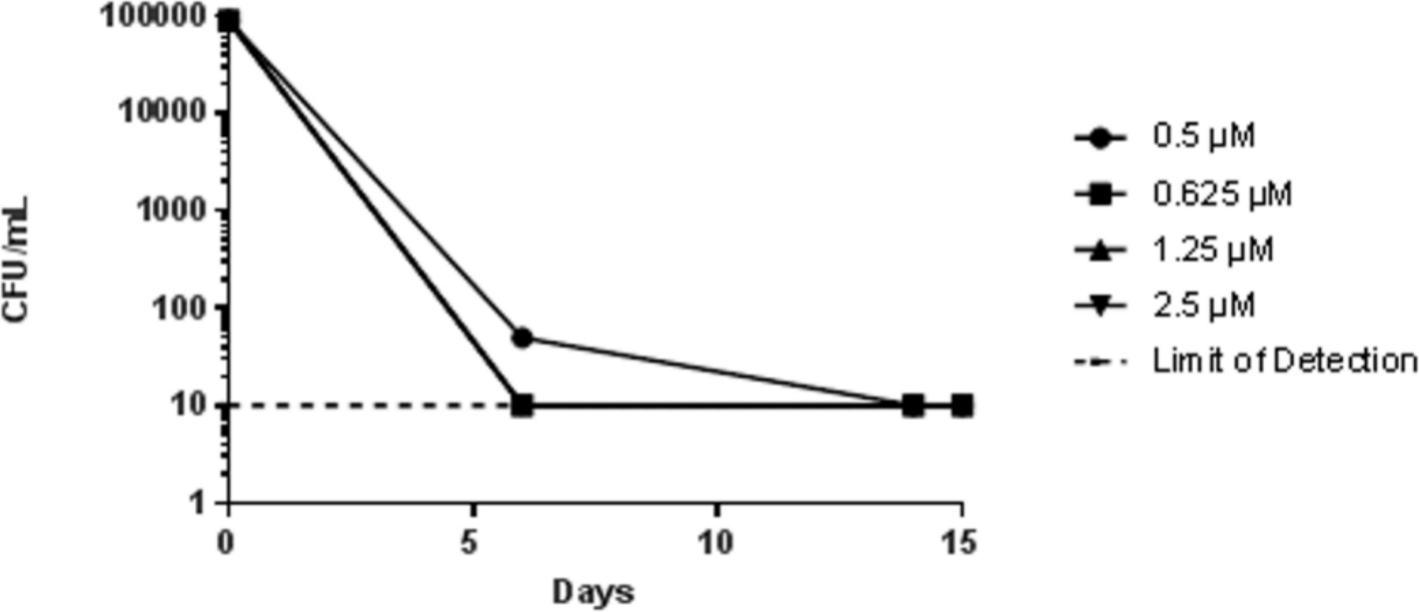
The representative aminothiazole 20 possesses bactericidal activity. *M*. *tuberculosis* was inoculated to a starting of OD_590_ of 0.1 in medium containing compound 20. CFU/mL was enumerated at indicated time points by serial dilution onto solid medium. The limit of detection was 20. Note that the lines for 0.625, 1.25 and 2.5 overlap.

We determined the effectiveness of a representative compound in killing aerobically-growing *M*. *tuberculosis*. Compound **20** had rapid killing activity, resulting in complete sterilization of cultures (>4 log kill) within 7 days (Fig 10), even at 0.625 μM. The minimum bactericidal concentration (MBC), defined as 3 log kill in 21 days, was less than the MIC i.e. < 0.5 μM, confirming that this compound is bactericidal (defined as MBC/MIC ratio <4).

### Lack of iron chelating activity

**Fig 11:**
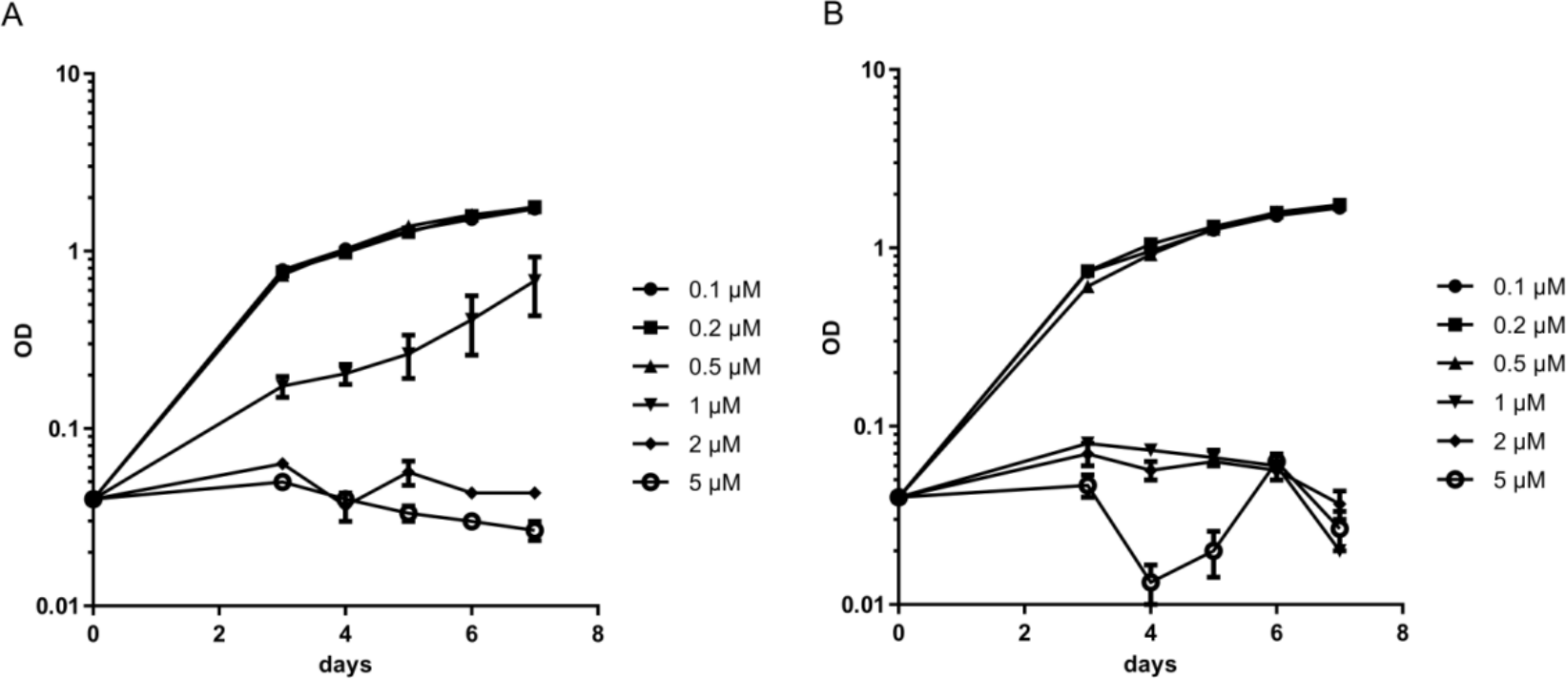
Inhibition of *M*. *tuberculosis* growth by AT compound 20. Growth curve in standard medium. (B) Growth curve in medium plus additional 0.04 g/L ferric ammonium citrate. Cells were inoculated to a starting OD_590_ of 0.04 into growth medium. Data are the mean +/- standard deviation of three independent cultures.

Due to the proximity of the heteroatomic pyridine and an electron-rich aminothiazole ring system, these aminothiazoles have the potential to chelate iron via the coordination of iron to the nitrogen atoms of both the pyridine and aminothiazole rings. This could be the mechanism of activity against *M*. *tuberculosis*, since iron is an essential nutrient for bacterial growth. To determine whether this is the case, we measured the capability of the representative compound **20** to inhibit growth in the presence of excess iron (Fig 11); we monitored growth over 7 days in six different concentrations bracketing the MIC in larger scale culture (growth tubes). As expected compound **20** completely prevented bacterial growth at the highest concentrations (2–5 μM); growth was also inhibited at 1 μM. When additional iron was provided there was no growth at concentrations of 1–5 μM, thus demonstrating that iron was not available to overcome compound-mediated inhibition and in fact if anything, the bacteria were slightly more susceptible.

We also tested iron supplementation in the form of hemin on solid medium with compound 20 and three other compounds, but no difference in MIC was seen (data not shown). This suggests that chelation of iron from the medium is not the mechanism of action for this class of compounds.

### Conclusions

Chemical library screening has successfully identified a number of compounds with excellent activity against *M*. *tuberculosis* including the 2-aminothiazoles [5]. We conducted an SAR assessment of different substitutions at the C-2 and C-4 positions, as well as possible replacements for the thiazole core. These modifications are in agreement with other aminothiazole analogs reported previously [10,11]. Concurrent with previous studies, a 2-pyridyl moiety at the C-4 position is essential for bacterial activity, as replacement of the pyridine ring resulted in a loss of activity. The efforts around the C-2 position indicate flexibility to various modifications with amine and amide all showing activity. 4-(Pyridin-2-yl)-*N*-(pyridin-3-yl)thiazol-2-amine (**20**) was the most potent analog prepared. Unfortunately, as with other active compounds presented in this manuscript and those reported previously [10,11], the activity and cytotoxicity of **20** are strongly correlated. However, a few analogs demonstrated improved selectivity. Amide-linked phenyl substituents at the C-2 position provided potent analogs with good separation of cytotoxicity, similar to what has been previously reported [10]. In this study, compound **33**, consisting of a 4-*tert*-butylbenzamide at the C-2 position, gave an MIC of 0.30 μM and a selectivity index of ~28 suggesting that cytotoxicity can be dialed out of the series. Urea-linkages at the C-2 position were not previously reported, but we demonstrated that they also have good potency and some selectivity. We also demonstrated that some ketones and carboxamide residues can confer moderate activity. This expands the opportunities for structure-property relationship (SPR) studies and property modulation for *in vivo* studies.

Representative compounds demonstrated selectivity towards mycobacteria, suggesting a target that is restricted to this genus. In kill kinetic studies, the representative compound **20** was bactericidal with rapid killing of the bacteria and complete culture sterilization in 7 days. These findings suggest targeting of a particularly vulnerable and essential enzyme or pathway in the bacterium. Although the mechanism of action for the compound series remains unknown, we confirmed that it is not likely to result from iron chelation in the medium. Thus, further development of the AT series is promising.

## Materials & Methods

### Determination of minimum inhibitory concentration (MIC)

MICs were determined against *M*. *tuberculosis* H37Rv (London Pride) [15] grown in Middlebrook 7H9 medium containing 10% OADC (oleic acid, albumin, dextrose, catalase) supplement (Becton Dickinson) and 0.05% w/v Tween 80 (7H9-Tw-OADC) under aerobic conditions. Bacterial growth was measured by OD after 5 days of incubation at 37°C. The MIC was defined as the minimum concentration at which growth was completely inhibited and was calculated from the inflection point of the fitted curve to the lower asymptote (zero growth).

### Cytotoxicity assay

The Vero cell line (African green monkey kidney epithelial cells: ATCC CRL-1587) was grown in DMEM, High Glucose, GlutaMAX™ (Invitrogen), 10% fetal bovine serum (FBS), and 1X penicillin-streptomycin solution (100 U/mL). Compounds were solubilized in DMSO and assayed as a 10-point three-fold serial dilution. Compounds were incubated with cells for 2 days at 37°C, 5% CO_2_. CellTiter-Glo® Reagent (Promega) was added and relative luminescent units (RLU) measured. Inhibition curves were fitted using the Levenberg–Marquardt algorithm. TC_50_ is the compound concentration that gave 50% inhibition of growth.

### Activity spectrum

The serial proportion method was used to determine MIC_99_ on solid medium [16]. LB was used for *E*. *coli* and *S*. *aureus*. Middlebrook 7H10 supplemented with 10% v/v OADC was used for mycobacteria. *E*. *coli* and *S*. *aureus* were incubated at 37°C overnight. *M*. *smegmatis* was incubated at 37°C for 3 days. The MIC_99_ was the lowest concentration of compound, which yielded less than 1% growth.

### Kill kinetics

A late log phase (OD_590_ 0.6–1.0) culture of *M*. *tuberculosis* (H37Rv) was adjusted to OD_590_ 0.1 in 7H9-Tw-OADC; 50 μL was used to inoculate 5 mL 7H9-Tw-OADC (10^5^ CFU/mL). Compound was added to each tube to a final DMSO concentration of 2%. Cultures were incubated at 37°C and serial dilutions plated on 7H10 agar plates to determine CFU/mL. Plates were incubated for 4 weeks before colonies were counted.

### Growth curves

Growth curves were conducted in 5 mL of 7H9-Tw-OADC in 16 x 125 mm glass tubes with stir bars. *M*. *tuberculosis* was inoculated to a theoretical starting OD_590_ of 0.04. OD_590_ was measured daily.

### Compound synthesis

^1^H and NMR spectral data were recorded in CDCl_3_ or DMSO-d_6_ on a 300 or 400 MHz Bruker NMR spectrometer. Column chromatography was conducted on Revelaris flash chromatography system. Reactions were monitored using thin-layer chromatography (TLC) on silica gel plates. HPLC analysis was conducted on an Agilent 1100 series LC system (Agilent ChemStation Rev.A.10.02; Phenomenex-Luna-C18, 4.8 mm × 150 mm, 5 μm, 1.0 mL/min, UV 254nm, room temperature) with MeCN/H_2_O (0.05% TFA or HCOOH buffer) gradient elution. HPLC-MS was performed on a Gilson 321 HPLC with detection performed by a Gilson 170 DAD and a Finnigan AQA mass spectrometer operating in electrospray ionization mode using a Phenomenex Gemini C18 150x4.6mm column. Compound purity was determined using an Agilent 1100 series LC system (Agilent ChemStation Rev.A.10.02; Phenomenex-Luna-C18, 4.8 mm × 150 mm, 5 μm, 1.0 mL/min, UV 254nm, room temperature) with MeCN/H_2_O (0.05% TFA or HCOOH buffer) gradient elution. All compounds were >95% pure via LC/MS analysis.

#### 2-Bromo-1-(pyridin-2-yl)ethan-1-one hydrobromide (3)

To a solution of 1-(pyridin-2-yl)ethan-1-one (3.0 g, 24.8 mmol) in HBr in AcOH (30 mL) at 0°C, bromine (3.9 g, 24.8 mmol) was added drop wise and allowed the mixture to stir at 70°C for 3 hours. After TLC showed completion, diluted the mixture with diethyl ether (50 mL) and filtered and washed with diethyl ether to obtain 2-bromo-1-(pyridin-2-yl)ethan-1-one hydrobromide **3** (6.5 g, 93% yield), as cream colored solid; ^1^H NMR (400 MHz, DMSO-d_6_): δ 5.02 (s, 2H), 7.73 (m, 1H), 8.01 (m, 2H), 8.75 (d, *J* = 4.4 Hz, 1H). All data are consistent with literature values [17].

#### 2-(Adamantane-1-carboxamido)-*N*-methoxy-*N*-methylthiazole-4-carboxamide (7)

To a solution of ethyl 2-aminothiazole-4-carboxylate (2.0 g, 11.63 mmol) in DCM (50 mL), adamantane-1-carboxylic acid (2.0 g, 11.63 mmol), PyBOP (18.1 g, 34.8 mmol), DIPEA (5 mL) and DMF (2 mL) were added and stirred at room temperature for 16 h. After completion, the reaction mixture was diluted with water (100 mL) and extracted with DCM (3 x 200 mL). The organic layer was dried over sodium sulfate and concentrated. The resulting crude was purified by column chromatography on silica gel (100–200 mesh) using 30% EtOAc in hexane as eluent to obtain **7** as a colorless oil (3.2 g, 84.2%). ^1^H NMR (400 MHz, MeOD): δ 7.94 (s, 1H), 4.36 (q, *J* = 7.2 Hz, 2H), 2.09 (m, 3H), 2.00 (s, 6H), 1.82 (m, 6H), 1.38 (t, *J* = 7.2 Hz, 3H).

MS m/z (M+H) 335.25.

#### *N*-(2,6-Dimethylphenyl)-2-picolinoylhydrazine-1-carbothioamide (10)

To a solution of picolinohydrazide (500 mg, 3.65 mmol) in ethanol (10 mL), 2-isothiocyanato-1,3-dimethylbenzene (595 mg, 3.65 mmol) was added and the mixture was refluxed for 15 h. After TLC showed completion, distilled off the solvent and the residue was washed with diethyl ether to obtain **10** (1.0 g, 91% yield), as off-white solid, which was used without further purification; MS m/z (M+H) 301.23.

#### 6-Chloro-*N*-(2,6-dimethylphenyl)pyrimidin-4-amine (12)

To a solution of 4,6-dichloropyrimidine **11** (1.0 g, 6.0 mmol) and 2,6-dimethylaniline (0.81 g, 6.0 mmol) in isopropanol (5 mL) in seal tube, catalytic HCl (0.1 mL) was added and heated the sealed contents at 100°C for 16 hours. After TLC showed completion, reaction mixture was concentrated under vacuum. The residue was diluted with water (5 mL) and extracted with EtOAc (3 x 20 mL). The organic layer was dried over Na_2_SO_4_ and concentrated. The resulting residue was purified by flash column chromatography and the desired product was eluted with 5% EtOAc in hexane. Concentration of the pure fractions afforded **12** (400 mg, 25% yield), as a light-brown solid; ^1^H NMR (400 MHz, DMSO-d_6_): δ 2.20 (s, 6H), 5.90 (m, 1H), 6.71 (brs, 1H), 7.26–7.16 (m, 2H), 7.36 (s, 1H), 8.42 (s, 1H); LCMS m/z (M+H) 234.27, purity: 97.1%.

#### 1-(2-Pyridyl)-*1H*-pyrazole-3-amine (15)

To *t*-BuOK (12 mL, 12 mmol) in *t*-BuOH (50 mL) was added 2-hydrazinopyridine (1.09 g, 10 mmol). To the mixture was added methoxyacrylonitrile (0.84 mL, 10 mmol) in *t*-BuOH (10 mL). The mixture was stirred under reflux conditions and the reaction was monitored via LC/MS. After 3 h, the reaction was cooled to room temperature and the solvent was removed. The resulting crude was suspended in water. The aqueous layer was extracted using EtOAc (3 x 50 mL). The organic layers were collected, dried over Na_2_SO_4_ and concentrated *in vacuo*. The resulting crude was purified via flash column chromatography to give **15** (113 mg, 7% yield), as yellow solid; ^1^H NMR: (300 MHz, CD_3_OD): δ 5.25 (s, 2H), 5.79–5.80 (m, 1H), 7.11–7.15 (m, 1H), 7.57 (d, *J* = 8.2 Hz, 1H), 7.82–7.88 (m, 1H), 8.25 (d, *J* = 3.5 Hz, 1H), 8.31–8.33 (m, 1H). All data are consistent with literature values [17,18].

#### 2-Amino-4-(pyridin-2-yl)thiazole (16)

To 2-bromo-1-pyridin-2-yl-ethanone (1 g, 5 mmol) in ethanol was added thiourea (340 mg, 5.1 mmol). The mixture was stirred at 70°C. The reaction was monitored via LC/MS. After 2 h, the reaction mixture was cooled to room temperature and precipitate was formed. The precipitate was collected by vacuum filtration and washed with acetone. The solid was dissolved in 2 M NaOH (25 mL) and extracted with EtOAc (3 x 50 mL). The combined organic layers were dried over Na_2_SO_4_ and concentrated *in vacuo* to give 3 (497 mg, 56% yield), as an off-white solid. ^1^H NMR: (300 MHz, CD_3_OD): δ 7.24–7.31 (m, 2H), 7.80–7.92 (m, 2H), 8.49–8.51 (m, 1H); ^13^C NMR: (300 MHz, CD_3_OD): δ 105.9, 120.8, 122.3, 137.3, 148.5, 149.5, 152.4; HRMS MS ESI *m/z* calcd for C_8_H_7_N_3_S (M+H)^+^ 178.0433, found 178.0441 (Δ 0.8 ppm). All data are consistent with literature values [19].

#### General synthesis (procedure I) for compounds 17–29 and 43–61

Compounds **17**–**29** and **43–61** were prepared following this general protocol unless otherwise noted. To substituted 2-bromoethanone in ethanol was added substituted thiourea (1.02 eq). The mixture was stirred at 70°C. The reaction was monitored via LC/MS. After 2 h, the reaction mixture was cooled to room temperature and precipitate was formed. The precipitate was collected by vacuum filtration and washed with acetone. The solid was dissolved in 2 M NaOH (25 mL) and extracted with EtOAc (3 x 50 mL). The combined organic layers were dried over Na_2_SO_4_ and concentrated *in vacuo* desired product.

#### *N*-(2,6-Difluorophenyl)-4-(pyridin-2-yl)thiazol-2-amine (17)

23 mg, 79% yield, as white solid; ^1^H NMR: (300 MHz, CDCl_3_): δ 6.99–7.05 (t, *J* = 8.1 Hz), 7.16–7.28 (m, 3H), 7.44 (s, 1H), 7.67–7.72 (m, 1H), 7.91 (d, *J* = 7.9 Hz, 1H), 8.56–8.58 (s, 1H); ^13^C NMR: (300 MHz, CDCl_3_): δ 107.9, 111.93, 111.99, 112.0, 112.2, 112.3, 120.8, 122.4, 126.5, 126.6, 126.8, 136.8, 149.3; HRMS MS ESI *m/z* calcd for C_14_H_9_F_2_N_3_S (M+H)^+^ 290.0558, found 290.0543 (Δ 1.5 ppm).

#### *N*-(2-Trifluoromethyl)-4-(pyridin-2-yl)thiazol-2-amine (18)

19 mg, 59% yield, as white solid; ^1^H NMR: (300 MHz, CDCl_3_): δ 7.12–7.28 (m, 2H), 7.41 (br s, 1H), 7.58–7.68 (m, 2H), 7.74–7.80 (m, 1H), 8.00 (d, *J* = 7.9 Hz, 1H), 8.19 (d, *J* = 8.3 Hz, 1H), 8.62–8.64 (m, 1H); ^13^C NMR: (300 MHz, CDCl_3_): δ 107.6, 120.3, 121.0, 122.6, 122.8, 126.7, 126.8, 133.24, 133.25, 136.9, 149.4, 163.6; HRMS MS ESI *m/z* calcd for C_16_H_15_N_3_S (M+H)^+^ 322.062, found 322.0602 (Δ 1.8 ppm).

#### *N*-(2,6-Dimethylphenyl)-4-(pyridin-2-yl)thiazol-2-amine (19)

16 mg, 57% yield, as beige crystals; ^1^H NMR: (300 MHz, CDCl_3_): δ 1.60 (s, 6H), 2.35 (s, 6H), 6.80 (br s, 1H), 7.17–7.24 (m, 5H), 7.69–7.74 (m, 1H), 7.90 (d, *J* = 7.9 Hz, 1H), 8.60–8.61 (m, 1H); ^13^C NMR: (300 MHz, CDCl_3_): δ 18.2, 106.1, 120.6, 122.3, 128.1, 136.7, 149.4; HRMS MS ESI *m/z* calcd for C_16_H_15_N_3_S (M+H)^+^ 282.1059, found 282.1039 (Δ 2 ppm).

#### 4-(Pyridin-2-yl)-*N*-(pyridin-3-yl)thiazol-2-amine (20)

To a solution of 1-(pyridin-3-yl)thiourea (0.2 g, 1.3 mmol) in DMF (6 mL), 2-bromo-1-(pyridin-2-yl)ethan-1-one hydrobromide **3** (0.55 g, 1.95 mmol) and triethylamine (0.53 mL, 3.9 mmol) were added successively and heated the mixture at 70°C for 2 hours. After TLC showed completion, reaction mixture was diluted with EtOAc (20 mL) and washed with water (3 x 10 mL). The organic layer was dried over Na_2_SO_4_ and concentrated. The resulting residue was purified by column chromatography (silicagel, 100–200#) and the desired product was eluted with 2.5% CH_3_OH in CH_2_Cl_2_. Concentration of the pure fractions afforded **20** (230 mg, 68% yield), as a white solid; ^1^H NMR: (400 MHz, DMSO-d_6_): δ 7.31–7.34 (1H, m), 7.38–7.41 (1H, m), 7.613 (1H, s), 7.88–7.93 (1H, m), 8.02 (1H, d, *J* = 7.6 Hz), 8.18–8.20 (1H, m), 8.30–8.33 (1H, m), 8.58 (1H, d, *J* = 4 Hz), 8.85 (1H, d, *J* = 2.4 Hz), 10.53 (1H, s); ^13^C NMR: (300 MHz, CDCl_3_): δ 110.0, 110.4, 116.4, 120.5, 122.4, 136.8, 137.7, 146.8, 149.5; LCMS m/z (M+H) 255.04, purity 99.9%; HRMS MS ESI *m/z* calcd for C_13_H_10_N_4_S (M+H)^+^ 255.0699, found 255.0681 (Δ 1.8 ppm).

#### *N*-(Phenyl)-4-(pyridin-2-yl)thiazol-2-amine (21)

13 mg, 52% yield, as beige crystals; ^1^H NMR: (300 MHz, CDCl_3_): δ 7.08–7.13 (m, 1H), 7.20–7.24 (m, 1H), 7.36–7.44 (m, 6H), 7.74–7.79 (m, 1H), 8.00 (d, *J* = 8.0 Hz, 1H), 8.62–8.63 (m, 1H); ^13^C NMR: (300 MHz, CDCl_3_): δ 106.3, 118.1, 120.9, 122.5, 123.0, 129.5, 136.9, 149.4; HRMS MS ESI *m*/*z* calcd for C_14_H_11_N_3_S (M+H)^+^ 254.0746, found 2 254.0734 54.0734 (Δ 1.2 ppm).

#### *N*-(Pyridin-3-yl)-4-(pyridin-2-yl)thiazol-2-amine (22)

15 mg, 62% yield, as white solid; ^1^H NMR: (300 MHz, CD_3_OD): δ 6.91–6.95 (m, 1H), 7.02–7.04 (m, 1H), 7.40 (s, 1H), 7.46–7.51 (m, 1H), 7.67–7.72 (m, 1H), 8.34 (m, 1H), 8.45–8.47 (m, 1H), 9.12 (br a, 1H); ^13^C NMR: (300 MHz, CD_3_OD): δ 107.1, 110.2, 115.8, 123.8, 133.7, 137.5, 146.2, 147.1; HRMS MS ESI *m/z* calcd for C_13_H_10_N_4_S (M+H)^+^ 255.0699, found 255.0678 (Δ 2.1 ppm).

#### *N*-(2-Methoxyphenyl)-4-(pyridin-2-yl)thiazol-2-amine (23)

To a solution of 1-(2-methoxyphenyl)thiourea (200 mg, 1.09 mmol) in DMF (6 mL), 2-bromo-1-(pyridin-2-yl)ethan-1-one hydrobromide **3** (460 mg, 1.64 mmol) and triethylamine (0.45 mL, 3.29 mmol) were added successively and heated the mixture at 70°C for 2 hours. After TLC showed completion, reaction mixture was diluted with EtOAc (20 mL) and washed with water (3 x 10 mL). The organic layer was dried over Na_2_SO_4_ and concentrated. The resulting residue was purified by column chromatography (silica gel, 100–200#) and the desired product was eluted with 20%EtOAc in hexane. Concentration of the pure fractions afforded **23** (170 mg, 55% yield), as a light-pink solid; ^1^H NMR: (400 MHz, DMSO-d_6_): δ 3.87 (s, 3H), 6.98–7.05 (m, 2H), 7.28–7.32 (m, 1H), 7.51 (s, 1H), 7.86–7.90 (m, 1H), 7.97 (d, *J* = 7.6 Hz, 1H), 8.51–8.54 (m, 1H), 8.56–8.58 (m, 1H), 9.75 (s, 1H); ^13^C NMR: (300 MHz, CDCl_3_): δ 55.7, 106.3, 110.1, 116.2, 121.0, 121.0, 121.1, 121.9, 122.4, 129.9, 136.8, 147.3, 149.3, 151.2, 152.7, 163.5; LCMS m/z (M+H) 284.08, purity 99.9%; HRMS MS ESI *m/z* calcd for C_15_H_13_N_3_OS (M+H)^+^ 284.0852, found 284.084 (Δ 1.2 ppm).

#### 4-(Pyridin-2-yl)-*N*-(pyridin-4-yl)thiazol-2-amine(24)

14 mg, 58% yield, as off-white solid; ^1^H NMR: (400 MHz, DMSO-d_6_): δ 7.32–7.36 (m, 1H), 7.68–7.70 (m, 3H), 7.90–7.94 (m, 1H), 8.05 (d, *J* = 7.6 Hz, 1H), 8.42 (d, *J* = 6.4 Hz, 1H), 8.60 (d, *J* = 4 Hz, 1H), 10.80 (1H, s); ^13^C NMR: (300 MHz, DMSO-d_6_): δ 109.0, 111.6, 120.9, 123.3, 137.8, 149.9, 150.7, 151.0, 152.3, 162.7; LCMS m/z (M+H) 255.04, purity 99.4%; HRMS MS ESI *m/z* calcd for C_13_H_10_N_4_S (M+H)^+^ 255.0699, found 255.0674 (Δ 2.5 ppm).

#### *N*-(Naphthalen-1-yl)-4-(pyridin-2-yl)thiazol-2-amine (25)

To a solution of 1-(naphthalen-1-yl)thiourea (0.2 g, 0.98 mmol) in DMF (10 mL), 2-bromo-1-(pyridin-2-yl)ethan-1-one hydrobromide **3** (0.416 g, 1.48 mmol) and triethylamine (0.41 mL, 2.96 mmol) were added successively and heated the mixture at 70°C for 2 hours. After TLC showed completion, reaction mixture was diluted with EtOAc (20 mL) and washed with water (3 x 10 mL). The organic layer was dried over Na_2_SO_4_ and concentrated. The resulting residue was purified by column chromatography (silica gel, 230–400#) and the desired product was eluted with 3% acetone in CH_2_Cl_2_. Concentration of the pure fractions afforded **25** (30 mg, 10% yield), as a brown solid; ^1^H NMR: (400 MHz, DMSO-d_6_): δ 7.29–7.32 (m, 1H), 7.53–7.59 (m, 4H), 7.67 (d, *J* = 8 Hz, 1H), 7.86–7.90 (m, 1H), 7.93–7.98 (m, 2H), 8.31–8.34 (m, 1H), 8.36 (d, *J* = 7.6 Hz, 1H), 8.59 (d, *J* = 4.8 Hz, 1H), 10.12 (s, 1H); ^13^C NMR: (300 MHz, DMSO-d_6_): δ 107.7, 116.6, 120.8, 122.4, 122.5, 123.0, 126.1, 126.2, 126.6, 128.7, 134.4, 137.0, 137.6, 149.8, 150.6, 162.6, 165.9; LCMS m/z (M+H) 304.18, purity 97.2%. HRMS MS ESI *m/z* calcd for C_18_H_13_N_3_S (M+H)^+^ 304.0903, found 304.089 (Δ 1.3 ppm).

#### *N*-(4-*tert*-Butylphenyl)-4-(pyridin-2-yl)thiazol-2-amine (26)

136 mg, 40% yield, as white solid; ^1^H NMR: (300 MHz, CDCl_3_): δ 1.34 (s, 9H), 7.19–7.23 (m, 1H), 7.33–7.41 (m, 5H), 7.61 (br s, 1H), 7.72–7.76 (m, 1H), 7.99–8.02 (m, 1H), 8.61–8.62 (m, 1H); ^13^C NMR: (300 MHz, CDCl_3_): δ 31.4, 106.0, 118.2, 120.9 121.0, 122.4, 122.5, 126.3, 136.8, 137.7, 149.3; LCMS m/z (M+H) 310.13, purity 99.9%; HRMS MS ESI *m/z* calcd for C_18_H_19_N_3_S (M+H)^+^ 310.1372, found 310.1359 (Δ 1.3 ppm).

#### *N*-Cyclohexyl-4-(pyridin-2-yl)thiazol-2-amine (27)

To a solution of 1-cyclohexylthiourea (0.227 g, 1.44 mmol) in DMF (7 mL), 2-bromo-1-(pyridin-2-yl)ethan-1-one hydrobromide **3** (0.350 g, 1.25 mmol) and triethylamine (0.35 mL, 2.50 mmol) were added successively and heated the mixture at 70°C for 2 hours. After TLC showed completion, reaction mixture was diluted with EtOAc (20 mL) and washed with water (3 x 10 mL). The organic layer was dried over Na_2_SO_4_ and concentrated. The resulting residue was purified by column chromatography (silica gel, 100–200#) and the desired product was eluted with 5% CH_3_OH in CH_2_Cl_2_. Concentration of the pure fractions afforded **27** (150 mg, 46% yield), as a cream colored solid; ^1^H NMR: (400 MHz, DMSO-d_6_): δ 1.20–1.36 (m, 5H), 1.54–1.57 (m, 1H), 1.68–1.72 (m, 2H), 1.97–2.00 (m, 2H), 3.47–3.53 (m, 1H), 7.22–7.26 (m, 2H),7.63 (d, *J* = 7.6 Hz, 1H), 7.79–7.86 (m, 2H), 8.53 (d, *J* = 6 Hz, 1H); ^13^C NMR: (300 MHz, CDCl_3_): δ 24.7, 25.5, 33.0, 55.0, 104.9, 120.8, 122.2, 136.7, 149.3, 152.9, 168.8; LCMS m/z (M+H) 260.09, purity 99.5%; HRMS MS ESI *m/z* calcd for C_14_H_17_N_3_S (M+H)^+^ 260.1216, found 260.1201 (Δ 1.5 ppm).

#### 4-(Pyridin-2-yl)-*N*-(4-(trifluoromethyl)phenyl)thiazol-2-amine (28)

To a solution of 1-(4-(trifluoromethyl)phenyl)thiourea (0.270 g, 1.23 mmol) in DMF (8 mL), 2-bromo-1-(pyridin-2-yl)ethan-1-one hydrobromide **3** (0.300 g, 1.07 mmol) and triethylamine (0.3 mL, 2.14 mmol) were added successively and heated the mixture at 70°C for 2 hours. After TLC showed completion, reaction mixture was diluted with EtOAc (20 mL) and washed with water (3 x 10 mL). The organic layer was dried over Na_2_SO_4_ and concentrated. The resulting residue was purified by column chromatography (silica gel, 100–200#) and the desired product was eluted with 30% EtOAc in hexane. Concentration of the pure fractions afforded **28** (65 mg, 19% yield), as a cream colored solid; ^1^H NMR: (400 MHz, DMSO-d_6_): δ 7.33–7.35 (m, 1H), 7.65 (s, 1H), 7.71 (d, *J* = 8.8 Hz, 2H), 7.88–7.95 (m, 3H), 8.05 (d, *J* = 8 Hz, 1H), 8.59–8.60 (m, 1H), 10.75 (s, 1H); ^13^C NMR: (300 MHz, DMSO-d_6_): δ 108.4, 117.0, 120.9, 123.2, 126.7, 126.81, 126.87, 137.7, 144.7, 149.9, 150.8, 152.4, 163.1; LCMS m/z (M+H) 322.05, purity 99.5%; HRMS MS ESI *m/z* calcd for C_15_H_10_F_3_N_3_S (M+H)^+^ 322.062, found 322.0607 (Δ 1.3 ppm).

#### *N*-(Naphthalen-2-yl)-4-(pyridin-2-yl)thiazol-2-amine (29)

A mixture of 1-(naphthalen-2-yl)thiourea (110 mg, 0.54 mmol) in DMF (5 mL), 2-bromo-1-(pyridin-2-yl)ethan-1-one hydrobromide **3** (229 mg, 0.81 mmol) and triethylamine (0.22 mL, 1.6 mmol) were added successively and heated the mixture at 70°C for 2 hours. After TLC showed completion, reaction mixture was diluted with EtOAc (20 mL) and washed with water (3 x 10 mL). The organic layer was dried over Na_2_SO_4_ and concentrated. The resulting residue was purified by flash column chromatography and the desired product was eluted with 15.5% EtOAc in hexane. Concentration of the pure fractions afforded **29** (85 mg, 51% yield), as a yellow solid; ^1^H NMR: (300 MHz, DMSO-d_6_): δ 7.33–7.39 (m, 2H), 7.46–7.50 (t, *J* = 7.0Hz, 1H), 7.58–759 (m, 1H), 7.62 (s, 1H), 7.82–7.98 (m, 4H), 8.12 (d, *J* = 7.8 Hz, 1H), 8.55 (s, 1H), 8.60–8.62 (m, 1H), 10.58 (br s, 1H); ^13^C NMR: (300 MHz, DMSO-d_6_): δ 107.5, 112.0, 119.4, 120.9, 123.1, 124.2, 126.9, 127.5 127.9, 129.1, 129.2, 134.4, 137.8, 139.1, 149.9, 150.9, 152.5, 163.6; LCMS m/z (M+H) 304.07, purity 99.1%; HRMS MS ESI *m/z* calcd for C_18_H_13_N_3_S (M+H)^+^ 304.0903, found 3 304.089504.0895 (Δ 0.8 ppm).

#### General synthesis for compounds 30–40

Compounds **20**–**30** were prepared following this general procedure unless otherwise noted. To **5** dissolved in THF was added triethylamine (1.0 eq). The mixture was cooled in an ice-bath, and to the mixture was added substituted acid chloride (1.0 eq). The mixture was stirred at room temperature and the reaction was monitored via LC/MS. After 1 h, the reaction solvent was evaporated. The resulting crude was purified by recrystallization from ethanol or purified via flash column chromatography to give desired product.

#### 2,2-Dimethyl-*N*- [4-(pyridin-2-yl)-1,3-thiazol-2-yl]propanamide (30)

14 mg, 33% yield, as off-white solid; ^1^H NMR: (300 MHz, CDCl_3_): δ 1.37 (s, 9H), 7.21–7.28 (m, 1H), 7.65 (s, 1H), 7.73–7.78 (t, *J* = 7.7 Hz, 1H), 7.88 (d, *J* = 8.0 Hz, 1H), 8.65–8.66 (m, 1H), 9.00 (br s, 1H); ^13^C NMR: (300 MHz, CDCl_3_): δ 27.2, 111.9, 120.4, 122.6, 136.8, 149.6; HRMS MS ESI *m/z* calcd for C_13_H_15_N_3_OS (M+H)^+^ 262.1009, found 262.0998 (Δ 1.1 ppm).

#### *N*- [4-(Pyridin-2-yl)-1,3-thiazol-2-yl]acetamide (31)

16 mg, 34% yield, as off-white solid; ^1^H NMR: (300 MHz, CD_3_OD): δ 2.2517 (s, 3H), 7.32–7.36 (m, 1H), 7.74 (br s, 1H), 7.86–7.91 (m, 1H), 8.07 (d, *J* = 8.0 Hz, 1H), 8.55–8.56 (m, 1H); ^13^C NMR: (300 MHz, CD_3_OD): δ 21.1, 111.3, 121.0, 122.6, 137.4, 148.6; HRMS MS ESI *m/z* calcd for C_10_H_9_N_3_OS (M+H)^+^ 220.0539, found 220.0526 (Δ 1.3 ppm).

#### *N*- [4-(Pyridin-2-yl)-1,3-thiazol-2-yl]cyclopropanecarboxamide (32)

19 mg, 43% yield, as off-white solid; ^1^H NMR: (300 MHz, CDCl_3_): δ 0.62–0.67 (m, 2H), 1.06–1.09 (m, 2H), 1.29–1.35 (m, 1H), 7.23–7.27 (m, 1H), 7.71–7.79 (m, 1H), 8.64–8.65 (m, 1H), 11.4 (br s, 1H); ^13^C NMR: (300 MHz, CDCl_3_): δ 9.2, 14.9, 111.9, 120.3, 122.7, 137.0, 149.1, 149.7, 152.1, 159.6, 172.2; HRMS MS ESI *m/z* calcd for C_12_H_11_N_3_OS (M+H)^+^ 246.0696, found 246.0675 (Δ 2.1 ppm).

#### 2-(*tert*-Butyl)-*N*- [4-(pyridin-2-yl)-1,3-thiazol-2-yl]benzamide (33)

24 mg, 70% yield, as a white solid; ^1^H NMR: (300 MHz, DMSO-d_6_): δ 1.25 (s, 9H), 7.33–7.37 (t, *J* = 6.5 Hz, 1H), 7.56 (d, *J* = 8.3 Hz, 2H), 7.89 (s, 2H), 8.02–8.11 (m, 3H), 8.61–8.63 (m, 1H), 12.74 (br s, 1H); ^13^C NMR: (300 MHz, DMSO-d_6_): δ 19.1, 22.8, 26.5, 43.4, 63.5, 67.5, 109.2, 113.1, 114.9, 119.4, 123.7, 131.5; HRMS MS ESI *m/z* calcd for C_19_H_19_N_3_OS (M+H)^+^ 338.1249, found 373.1660 parent ion not detected.

#### 2-(Dimethylamino)-*N*- [4-(pyridin-2-yl)-1,3-thiazol-2-yl]acetamide (34)

16 mg, 39% yield, as brown solid; ^1^H NMR: (300 MHz, CDCl_3_): δ 7.69–7.37 (m, 1H), 8.18 (s, 1H), 8.29–8.39 (m, 2H), 8.69–8.71 (m, 1H); ^13^C NMR: (300 MHz, CDCl_3_): δ 43.3, 57.6, 116.1, 122.6, 124.2, 124.3, 142.3, 144.5, 144.6, 158.1, 163.5; HRMS MS ESI *m/z* calcd for C_12_H_14_N_4_OS (M+H)^+^ 263.0961, found 263.0937 (Δ 2.4 ppm).

#### *N*- [4-(Pyridin-2-yl)-1,3-thiazol-2-yl]cyclohexanecarboxamide (35)

21 mg, 48% yield, as white solid; ^1^H NMR: (300 MHz, CDCl_3_): δ 1.19–2.01 (m, 10H), 2.46–2.54 (m, 1H), 6.26 (br s, 3H), 7.54–7.59 (m, 1H), 7.85 (s, 1H), 8.07–8.18 (m, 2H), 9.04 (m, 1H); ^13^C NMR: (300 MHz, CDCl_3_): δ 25.3, 25.5, 29.0, 44.7, 116.2, 122.1, 123.9, 142.3, 144.8, 147.5, 160.2, 175.2; HRMS MS ESI *m/z* calcd for C_15_H_17_N_3_OS (M+H)^+^ 288.1165, found 288.1144 (Δ 2.1ppm).

#### 2-Oxo-2- [[4-(2-pyridinyl)-2-thiazolyl]amino]ethyl] ester acetic acid (36)

6 mg, 24% yield, as waxy white solid; ^1^H NMR: (300 MHz, DMSO-d_6_): δ 2.14 (s, 3H), 7.39 (m, 1H), 7.90–7.97 (m, 3H), 8.63 (m, 1H), 12.57 (s, 1H); HRMS MS ESI *m/z* calcd for C_12_H_11_N_3_O_3_S (M+H)^+^ 278.0594, found 278.0582 (Δ 1.2 ppm).

#### 2-Hydroxy-*N*- [4-(2-pyridinyl)-2-thiazolyl]acetamide (37)

To **36** (5 mg, 0.1 mmol) was added 5% K_2_CO_3_ in water (2 mL). The mixture was stirred at room temperature and the reaction was monitored via LC/MS. After 3 h, the aqueous layer was extracted with EtOAc (3 x 25 mL). The organic layers were collected, dried over Na_2_SO_4_ and concentrated *in vacuo*. The resulting crude was purified via flash column chromatography to give **37** (4 mg, 100% yield), as a white solid; ^1^H NMR: (300 MHz, DMSO-d_6_): δ 4.17 (s, 2H), 7.35–7.39 (m, 1H), 7.87–8.00 (m, 3H), 8.60–8.62 (m, 1H), 11.95 (br s, 1H); HRMS MS ESI *m/z* calcd for C_10_H_9_N_3_O_2_S (M+H)^+^ 236.0488, found 236.0500 (Δ 1.2 ppm).

#### 4-Nitro-*N*- [4-(2-pyridinyl)-2-thiazolyl]benzamide (38)

A solution of 4-nitrobenzoic acid (94 mg, 0.56 mmol) and PyBOP (440 mg, 0.84 mmol) and DIPEA (1.25 mL, 7.56 mmol) in CH_2_Cl_2_ (10 mL) was stirred for 15 minutes and then 4-(pyridin-2-yl)thiazol-2-amine (100 mg, 0.56 mmol) was added. The reaction mixture was then stirred at room temperature and monitored by TLC analysis (EtOAc:hexane = 1:1) until completion. After 16 h, the reaction mixture was poured to ice cooled water (30 mL) and extracted with CH_2_Cl_2_ (3 x 50 mL). The combined organic layer was dried over Na_2_SO_4_ and concentrated under reduced pressure. The crude was further purified via flash column chromatography on silica gel (100–200 mesh) using 2% CH_3_OH in CH_2_Cl_2_ as eluent to afford **38** (30 mg, 16% yield), as a yellow solid; ^1^H NMR: (300 MHz, DMSO-d_6_): δ 3.41 (s, 3H), 7.35–7.39 (m, 1H), 7.89–7.96 (m, 2H), 8.02 (d, *J* = 7.7 Hz, 1H), 8.33–8.41 (m, 4H), 8.63–8.64 (m, 1H), 13.21 (s, 1H); LCMS m/z (M+H) 327.09, purity: 96.6%; HRMS MS ESI *m/z* calcd for C_15_H_10_N_4_O_3_S (M+H)^+^ 327.0546, found 327.0525 (Δ 2.1 ppm).

#### 2-Hydroxy-*N*-(4-(pyridin-2-yl)thiazol-2-yl)benzamide (39)

(i) To a mixture of salicylic acid (1.30 g, 9.4 mmol) in acetic anhydride (2.80 g, 28.3 mmol) 3 drops of phosphoric acid was added and stirred the mixture at 90°C for 5 minutes. Cooled the mixture and filtered the solid that formed and dried under vacuum to obtain 2-acetoxybenzoic acid (1.20 g, 71% yield), as a white solid; ^1^H NMR (400 MHz, CDCl_3_): δ 8.12 (d, *J* = 7.6 Hz, 1H), 7.63 (t, *J* = 7.6 Hz, 1H), 7.36 (t, *J* = 7.6 Hz, 1H), 7.14 (d, *J* = 8.0 Hz, 1H), 2.35 (s, 3H). MS m/z (M-H) 179.05

(ii) A solution of 2-acetoxybenzoic acid (200 mg, 1.13 mmol) and PyBOP (810 mg, 1.69 mmol) and DIPEA (0.58 mL, 3.3 mmol) in CH_2_Cl_2_ (10 mL) was stirred for 15 minutes and then 4-(pyridin-2-yl)thiazol-2-amine **5** (200 mg, 1.13 mmol) was added. The reaction mixture was then stirred at room temperature for 16h. After TLC showed completion, the reaction mixture was diluted with CH_2_Cl_2_ (30 mL) and washed with water (2 x 20 mL). The organic layer was dried over Na_2_SO_4_ and concentrated. The resulting crude was purified by column chromatography on silica gel (100–200 mesh) using 2% CH_3_OH in CH_2_Cl_2_ as eluent to afford the de-acetylated product **39** (13 mg, 3% yield), as a white solid and de-acetylated product (40 mg, 12% yield); ^1^H NMR: (300 MHz, DMSO-d_6_): δ 1.24–1.27 (m, 1H), 7.00–7.09 (m, 2H), 7.36 (m, 1H), 7.48–7.53 (m, 1H), 7.87–7.92 (m, 2H), 8.01–8.04 (m, 2H), 8.62–8.63 (m, 1H), 11.76 (s, 1H), 12.06 (s, 1H); ^13^C NMR: (300 MHz, DMSO-d_6_): δ 112.7, 117.6, 119.9, 120.7, 123.4, 130.8, 135.0, 137.7, 149.9; LCMS m/z (M-H) 298.10, purity: 94.1%; HRMS MS ESI *m/z* calcd for C_15_H_11_N_3_O_2_S (M+H)^+^ 298.06055, found 298.0634 (Δ 2.8 ppm).

#### 3,5-Dinitro-*N*- [4-(2-pyridinyl)-2-thiazolyl]benzamide (40)

25 mg, 44% yield, as white solid; ^1^H NMR: (300 MHz, DMSO-d_6_): δ 7.38–7.42 (t, *J* = 6.3 Hz, 1H), 7.93–8.07 (m, 3H), 8.64–8.66 (m, 1H), 9.05 (s, 2H), 9.33 (s, 2H), 13.6 (s, 1H); HRMS MS ESI *m/z* calcd for C_15_H_9_N_5_O_5_S (M+H)^+^ 372.0397, found 372.0379 (Δ 1.8 ppm).

#### *N*-Cyclopentyl-*N’*- [4-(2-pyridinyl)-2-thiazolyl]urea (41)

To **5** (18 mg, 0.1 mmol) dissolved in THF (2 mL) was added cyclopentyl isocyante (0.011 mL, 0.1 mmol). The mixture was stirred at room temperature and the reaction was monitored via LC/MS. After 1 h, the reaction mixture was evaporated and the resulting crude was recrystallized from ethanol to give **41** (4 mg, 13% yield), as a white solid; ^1^H NMR: (300 MHz, DMSO-d_6_): δ 1.37–1.45 (m, 2H), 1.52–1.68 (m, 4H), 1.82–1.90 (m, 2H), 3.92–4.03 (m, 2H), 6.60–6.62 (m, 1H), 7.38–7.42 (m, 1H), 7.75 (s, 1H), 7.97–7.98 (m, 2H), 8.59–8.61 (m, 1H), 10.34 (s, 1H); HRMS MS ESI *m/z* calcd for C_14_H_16_N_4_OS (M+H)^+^ 289.1118, found 289.1114 (Δ 0.4 ppm).

#### *N*-Phenyl-*N'*- [4-(2-pyridinyl)-2-thiazolyl]urea (42)

To **5** (18 mg, 0.1 mmol) dissolved in THF (2 mL) was added phenyl isocyante (0.018 mL, 0.1 mmol). The mixture was stirred at room temperature and the reaction was monitored via LC/MS. After 1 h, the reaction mixture was evaporated and the resulting crude was recrystallized from ethanol to give **42** (30 mg, 100% yield), as a white solid. ^1^H NMR: (300 MHz, DMSO-d_6_): δ 7.03–7.08 (t, *J* = 7.3 Hz, 1H), 7.31–7.36 (t, *J* = 7.5 Hz, 2H), 7.40–7.50 (m, 3H), 7.86 (s, 1H), 7.98–8.05 (m, 2H), 8.62–8.63 (m, 1H), 9.01 (s, 1H), 10.75 (br s, 1H); ^13^C NMR: (300 MHz, DMSO-d_6_): δ 113.2, 119.1, 121.4, 123.3, 123.8, 129.3, 138.8, 140.0, 148.0, 152.0, 160.0; HRMS MS ESI *m/z* calcd for C_15_H_12_N_4_OS (M+H)+ 297.0805, found 297.0790 (Δ 1.5 ppm).

#### General synthesis for compounds 43–61

Compounds **43–61** were prepared following the general protocol (procedure I) described for compounds **17–29** above.

#### 4-Phenyl-*N*- [2-(trifluoromethyl)phenyl]-1,3-thiazol-2-amine (43)

43 mg, 64% yield, as pink solid; ^1^H NMR: (300 MHz, CDCl_3_): δ 6.93 (s, 1H), 7.16–7.21 (t, *J* = 7.5 Hz, 1H), 7.33–7.47 (m, 4H), 7.58–7.68 (m, 2H), 7.88 (d, *J* = 7.1Hz, 1H), 8.25 (d, *J* = 8.3 Hz, 1H); ^13^C NMR: (300 MHz, CDCl_3_): δ 103.2, 120.3, 122.7, 126.1, 126.6, 126.7, 128.0, 128.6, 133.2; HRMS MS ESI *m/z* calcd for C_16_H_11_F_3_N_2_S (M+H)^+^ 321.0668, found 321.0653 (Δ 1.5 ppm).

#### 4-(Thiophen-2-yl)-*N*- [2-(trifluoromethyl)phenyl]-1,3-thiazol-2-amine (44)

36 mg, 65% yield, as off-white solid; ^1^H NMR: (300 MHz, CDCl_3_): δ 6.79 (s, 1H), 7.06–7.09 (m, 1H), 7.17–7.22 (t, *J* = 7.6 Hz, 1H), 7.37–7.43 (m, 2H), 7.57–7.67 (m, 2H), 8.17 (d, *J* = 8.2 Hz, 1H); ^13^C NMR: (300 MHz, CDCl_3_): δ 101.8, 120.6, 123.0, 123.9, 125.0, 126.6, 126.70, 126.78, 126.8, 127.6, 133.2, 133.3; HRMS MS ESI *m/z* calcd for C_14_H_9_F_3_N_2_S_2_ (M+H)^+^ 327.0232, found 327.0210 (Δ 2.2 ppm).

#### 4-(Naphthalen-2-yl)-*N*- [2-(trifluoromethyl)phenyl]-1,3-thiazol-2-amine (45)

27 mg, 74% yield, as off-white solid; ^1^H NMR: (300 MHz, CDCl_3_): δ 7.04 (s, 1H), 7.18–7.23 (m, 1H), 7.47–7.55 (m, 2H), 7.60–7.70 (m, 2H), 8.24 (d, *J* = 8.2 Hz, 1H), 8.41 (s, 1H); ^13^C NMR: (300 MHz, CDCl_3_): δ 103.7, 120.5, 122.8, 124.0, 125.2, 126.1, 126.3, 126.7, 126.8, 127.7, 128.3, 128.4, 128.5, 133.3; HRMS MS ESI *m/z* calcd for C_20_H_13_F_3_N_2_S (M+H)^+^ 371.0824, found 371.0803 (Δ 2.1 ppm).

#### 4-(2-Methoxyphenyl)-*N*- [2-(trifluoromethyl)phenyl]-1,3-thiazol-2-amine (46)

30 mg, 70% yield, as white crystals; ^1^H NMR: (300 MHz, CDCl_3_): δ 3.98 (s, 3H), 7.00 (d, *J* = 8.2 Hz, 1H), 7.06–7.19 (dt, *J*_*1*_ = 21.8 Hz, *J*_*2*_ = 7.5 Hz, 2H), 7.28–7.35 (m, 2H), 7.44 (s, 1H), 7.57–7.66 (m, 2H), 8.19–8.22 (dd, *J*_*1*_ = 7.7 Hz, *J*_*2*_ = 1.5 Hz, 1H), 8.27 (d, *J* = 8.3 Hz, 1H); ^13^C NMR: (300 MHz, CDCl_3_): δ 55.4, 108.1, 111.0, 120.0, 120.8, 122.3, 126.5, 126.6, 128.7, 130.0, 133.2, 147.3, 156.9, 161.4; HRMS MS ESI *m/z* calcd for C_17_H_13_F_3_N_2_OS (M+H)^+^ 351.0773, found 351.0752 (Δ 2.1 ppm).

#### 2-(2- [2-(Trifluoromethyl)phenyl]amino-1,3-thiazol-4-yl)phenol (47)

37 mg, 67% yield, as white crystals; ^1^H NMR: (300 MHz, CDCl_3_): δ 6.87–6.92 (m, 2H), 6.98–7.01 (m, 1H), 7.22–7.25 (m, 1H), 7.30–7.32 (m, 1H), 7.57–7.71 (m, 3H), 7.90 (d, *J* = 8.2 Hz, 1H), 11.2 (br s, 1H); ^13^C NMR: (300 MHz, CDCl_3_): δ 101.7, 117.4, 117.7, 119.4, 121.9, 124.2, 125.8, 126.8, 126.9, 127.01, 127.08, 130.0, 133.3, 155.7; HRMS MS ESI *m/z* calcd for C_16_H_11_F_3_N_2_OS (M+H)^+^ 337.0617, found 337.0592 (Δ 2.5 ppm).

#### 4- [(3R,5S,7s)-Adamantan-1-yl]-N- [2-(trifluoromethyl)phenyl]-1,3-thiazol-2-amine (48)

53 mg, 73% yield, as white crystals; ^1^H NMR: (300 MHz, CDCl_3_): δ 1.79 (s, 6H), 1.98 (s, 6H), 2.10 (br s, 3H), 6.27 (s, 1H), 7.09–7.14 (t, *J* = 7.5 Hz, 1H), 7.52–7.57 (t, *J* = 8.0 Hz, 1H), 7.60 (d, *J* = 7.8 Hz, 1H), 8.17 (d, *J* = 8.2 Hz, 1H); ^13^C NMR: (300 MHz, CDCl_3_): δ 28.5, 36.8, 41.8, 100.8, 119.7, 122.0, 126.5, 126.6, 133.1; HRMS MS ESI *m/z* calcd for C_20_H_21_F_3_N_2_S (M+H)^+^ 379.145, found 379.1430 (Δ 0.2 ppm).

#### *N*-(2,6-Dimethylphenyl)-4-phenyl-1,3-thiazol-2-amine (49)

40 mg, 81% yield, as white solid; ^1^H NMR: (300 MHz, CDCl_3_): δ 2.35 (s, 6H), 6.63 (s, 1H), 7.15–7.20 (m, 6H), 7.64–7.67 (m, 2H), 8.64 (br s, 1H); ^13^C NMR: (300 MHz, CDCl_3_): δ 18.2, 101.2, 125.9, 127.5, 127.9, 128.3, 128.8, 134.7, 137.2, 137.7, 151.7, 170.9; HRMS MS ESI *m/z* calcd for C_14_H_11_N_3_S (M+H)^+^ 281.1107, found 281.1091 (Δ 1.6 ppm).

#### *N*-(2,6-Dimethylphenyl)-4-(thiophen-2-yl)-1,3-thiazol-2-amine (50)

46 mg, 83% yield, as white solid; ^1^H NMR: (300 MHz, CDCl_3_): δ 2.34 (s, 6H), 6.54 (s, 1H), 6.91–6.94 (t, *J* = 4.9 Hz, 1H), 7-14-7.24 (m, 5H), 7.89 (br s, 1H); ^13^C NMR: (300 MHz, CDCl_3_): δ 18.2, 100.3, 123.4, 124.3, 127.4, 128.1, 128.9, 137.2, 137.4, 138.6, 146.0, 170.5; HRMS MS ESI *m/z* calcd for C_15_H_14_N_2_S_2_ (M+H)^+^ 287.0671, found 287.0655 (Δ 1.6 ppm).

#### *N*-(2,6-Dimethylphenyl)-4-(pyridin-3-yl)-1,3-thiazol-2-amine (51)

34 mg, 40% yield, as off-white solid; ^1^H NMR: (300 MHz, CDCl_3_): δ 2.34 (s, 5H), 6.73 (s, 1H), 7.92–7.94 (m, 1H), 8.44 (d, *J* = 42.6 Hz, 2H), 9.10 (s, 1H); ^13^C NMR: (300 MHz, CDCl_3_): δ 18.1, 102.6, 123.1, 128.0, 128.9, 133.1, 137.0, 147.4, 148.3, 171.1; LCMS m/z (M+H) 283.08, purity 99.81%; HRMS MS ESI *m/z* calcd for C_16_H_15_N_3_S (M+H)^+^ 282.1059, found 282.1045 (Δ 1.4 ppm).

#### *N*-(2,6-Dimethylphenyl)-4-(pyrazin-2-yl)thiazol-2-amine (52)

To a solution of 1-(pyrazin-2-yl)ethan-1-one (250 mg, 2.03 mmol) in AcOH (5 mL), pyridinium bromide perbromide (780 mg, 2.4 mmol) was added and allowed the mixture to stir at room temperature for 48 hours. After TLC showed completion, diluted the mixture with EtOAc (20 mL) and washed with water (3 x 10 mL). The organic layer was dried over Na_2_SO_4_ and concentrated to obtain crude 2-bromo-1-(pyrazin-2-yl)ethan-1-one, which was used in next step without further purification (300 mg, crude). To a solution of 1-(2,6-dimethylphenyl)thiourea (134 mg, 0.7 mmol) in DMF (5 mL), crude 2-bromo-1-(pyrazin-2-yl)ethan-1-one (300 mg, 0.7 mmol) and triethylamine (0.31 mL, 2.2 mmol) were added successively and heated the mixture at 70°C for 2 hours. After TLC showed completion, reaction mixture was diluted with EtOAc (20 mL) and washed with water (3 x 10 mL). The organic layer was dried over Na_2_SO_4_ and concentrated. The resulting residue was purified by column chromatography (silicagel, 100–200#) and the desired product was eluted with 20% EtOAc in hexane. Concentration of the pure fractions afforded **52** (135 mg, 32% yield), as a pale yellow solid; ^1^H NMR: (400 MHz, DMSO-d_6_): δ 2.23 (s, 6H), 7.14–7.20 (m, 3H), 7.45 (s, 1H), 8.54 (d, *J* = 2.4 Hz, 1H), 8.61 (t, *J* = 1.6 Hz, 1H), 8.99 (d, *J* = 1.6 Hz, 1H), 9.43 (1H, s); ^13^C NMR: (300 MHz, CDCl_3_) δ 18.1, 107.7, 128.1, 128.9, 137.0, 137.4, 142.5, 142.7, 143.8, 148.3, 148.8, 171.0; LCMS m/z (M+H) 283.08, purity 99.8%; HRMS MS ESI *m/z* calcd for C_15_H_14_N_4_S (M+H)^+^ 283.1012, found 283.1001 (Δ 1.1 ppm).

#### *N*-(2,6-Dimethylphenyl)-4-(pyrimidin-4-yl)thiazol-2-amine (53)

(i) To a suspension of 4-methylpyrimidine (5.0 g, 0.053 mol) in water (300 mL), KMNO_4_ (21 g, 0.132 mol) and KOH (20.8 g, 0.372 mol) were added and heated the mixture at 75°C for 1.5 hours. Cooled to room temperature and ethanol (300 mL) was added drop wise. Filtered off the solids over Celite and concentrated the filtrate. The resulting crude was diluted with water (50 mL) and acidified with conc. HCl. Precipitate was collected and dried under vacuum to obtain pyrimidine-4-carboxylic acid (4.5 g, 67% yield), as a white solid. ^1^H NMR (400 MHz, DMSO-d_6_): δ 13.93 (brs, 1H), 9.37 (d, *J* = 1.2HZ, 1H), 9.06 (d, J = 4.8 Hz, 1H), 8.01 (dd, *J* = 4.8, 1.2 Hz, 1H).

(ii) To a solution of pyrimidine-4-carboxylic acid (2.0 g, 16.0 mmol) in THF (25 mL), EDC.HCl (3.1 g, 16.5 mmol), HOBt (1.5 g, 10 mmol) and DIPEA (2.5 mL, 13.7 mmol) were sequentially added. After stirring this mixture for 1 hour under N_2_ atmosphere, *N*,*O*-dimethylhydroxylamine hydrochloride (1.57 g, 16.0 mmol) was added continued stirring for 16 h. After completion, distilled off the solvent and diluted the residue with water (50 mL). The aqueous layer was extracted with EtOAc (3 x 100 mL), the combined organic layer was dried over anhydrous Na_2_SO_4_ and concentrated under reduced pressure. The crude was further purified by column chromatography on silica gel (100–200 #) using 25% EtOAc in hexane as eluent to afford *N*-methoxy-*N*-methylpyrimidine-4-carboxamide (2.4 g, 89% yield), as a viscous liquid.

(iii) To a solution of *N*-methoxy-*N*-methylpyrimidine-4-carboxamide (2.25 g, 13.4 mmol) in dry THF (15 mL) at -55°C under nitrogen atmosphere, methyl magnesium bromide (5.4 mL, 3 M solution, 16.1 mmol) was added drop wise and allowed the reaction to reach room temperature. After TLC showed completion, quenched the reaction with saturated ammonium chloride solution (10 mL) and extracted with EtOAc (3 x 25 mL). The combined organic layer was dried over anhydrous Na_2_SO_4_ and concentrated under reduced pressure. The crude was purified by column chromatography on silica gel (100–200 #) using 5% EtOAc in hexane as eluent to afford 1-(pyrimidin-4-yl)ethan-1-one (225 mg, 14% yield), as a white solid ^1^H NMR (400 MHz, DMSO-d_6_): δ 9.42 (s, 1H), 9.09 (d, *J* = 4.8 Hz, 1H), 7.90 (dd, J = 5.2, 1.2 Hz, 1H), 2.65 (s, 3H).

(iv) To a solution of 1-(pyrimidin-4-yl)ethan-1-one (0.30 g, 2.4 mmol) in AcOH (10 mL), pyridinium bromide perbromide (0.94 g, 2.9 mmol) was added and allowed the mixture to stir at room temperature for 48 hours. After TLC showed completion, diluted the mixture with EtOAc (30 mL) and washed with water (3 x 20 mL). The organic layer was dried over Na_2_SO_4_ and concentrated to obtain crude 2-bromo-1-(pyrazin-2-yl)ethan-1-one (510 mg, crude), which was used in next step without further purification.

(v) To a solution of 1-(2,6-dimethylphenyl)thiourea (179 mg, 0.99 mmol) in DMF (10 mL), 2-bromo-1-(pyrimidin-4-yl)ethan-1-one (200 mg, 0.99 mmol) and triethylamine (0.4 mL, 3 mmol) were added successively and heated the mixture at 70°C for 2 hours. After TLC showed completion, reaction mixture was diluted with EtOAc (30 mL) and washed with water (3 x 10 mL). The organic layer was dried over Na_2_SO_4_ and concentrated. The resulting residue was purified by column chromatography (silicagel, 100–200#) and the desired product was eluted with 25% EtOAc in hexane. Concentration of the pure fractions afforded **53** (110 mg, 39% yield), as a pale yellow solid; ^1^H NMR: (300 MHz, CDCl_3_): δ 2.34 (s, 6H), 7.17–7.23 (m, 3H), 7.48 (s, 1H), 7.63–7.64 (m, 1H), 8.18 (br s, 1H), 8.54–8.56 (m, 1H), 9.00 (br s, 1H); ^13^C NMR: (300 MHz, CDCl_3_): δ 18.1, 110.2, 116.8, 128.3, 129.0, 137.0, 157.5, 158.7; LCMS m/z (M+H) 283.08, purity 99.8%; HRMS MS ESI *m/z* calcd for C_15_H_14_N_4_S (M+H)^+^ 283.1012, found 283.1001 (Δ 1.1 ppm).

#### *N*-(2,6-Dimethylphenyl)-4-(*1H*-pyrazol-3-yl)thiazol-2-amine (54)

To a solution of 1-(2,6-dimethylphenyl)thiourea (0.666 g, 3.70 mmol) in DMF (10 mL), 2-bromo-1-(1H-pyrazol-3-yl)ethan-1-one (0.70 g, 3.70 mmol) and triethylamine (1 mL, 7.40 mmol) were added successively and heated the mixture at 70°C for 2 hours. After TLC showed completion, the reaction mixture was diluted with EtOAc (30 mL) and washed with water (3 x 10 mL). The organic layer was dried over Na_2_SO_4_ and concentrated. The resulting residue was purified by column chromatography (silicagel, 100–200#) and the desired product was eluted with 50% EtOAc in hexane. Concentration of the pure fractions afforded **54** (215 mg, 21% yield), as an off-white solid; ^1^H NMR: (300 MHz, DMSO-d_6_): δ 2.22 (s, 6H), 6.44 (s, 1H), 6.83 (m, 1H), 7.15 (s, 3H), 7.47–7.69 (m, 1H), 9.21 (s, 1H); LCMS m/z (M+H) 271.11, purity: 97.9%; HRMS MS ESI *m/z* calcd for C_14_H_14_N_4_S (M+H)^+^ 271.1012, found 271.0998 (Δ 1.4 ppm).

#### *N*-(4-Phenyl-1,3-thiazol-2-yl)pyridin-2-amine (55)

To 4-phenyl-2-thiazolamine (50 mg, 0.3 mmol) dissolved in DMF (2 mL) was added K_2_CO_3_ (78 mg, 0.6 mmol), followed by the addition of 2-chloropyridine (0.02 mL, 0.3 mmol). The mixture was stirred at 90°C and the reaction was monitored via LC/MS. After 16 h, the reaction was cooled to room temperature, diluted with water and extracted with EtOAc (3 x 25 mL). The combined organic layers were washed with brine (5 x 25 mL), collected, dried over Na_2_SO_4_ and concentrated *in vacuo*. The resulting crude was purified via flash column chromatography to give **55** (40 mg, 81% yield), as an white solid. ^1^H NMR: (300 MHz, CDCl_3_): δ 6.31 (d, *J* = 8.3 Hz, 1Hz), 6.76–6.80 (t, *J* = 6.6 Hz, 1H), 7.10 (s, 1H), 7.22–7.32 (m, 2H), 7.36–7.41 (t, *J* = 7.6 Hz, 2H), 7.94 (d, *J* = 7.2 Hz, 2H), 8.33–8.34 (m, 1H), 11.2 (s, 1H); ^13^C NMR: (300 MHz, CDCl_3_): δ 105.7, 110.8, 116.1, 126.2, 127.8, 128.7, 134.8, 137.2, 146.4, 149.3, 151.4, 161.7; HRMS MS ESI *m/z* calcd for C_14_H_15_F_3_N_2_S (M+H)^+^ 254.0746, found 254.0731 (Δ 1.5 ppm).

#### *N*- [4-(Thiophen-2-yl)-1,3-thiazol-2-yl]pyridin-2-amine (56)

To 4-(2-thienyl)-2-thiazolamine (50 mg, 0.3 mmol) dissolved in DMF (2 mL) was added K_2_CO_3_ (78 mg, 0.6 mmol), followed by the addition of 2-chloropyridine (0.02 mL, 0.3 mmol). The mixture was stirred at 90°C and the reaction was monitored via LC/MS. After 16 h, the reaction was cooled to room temperature, diluted with water and extracted with EtOAc (3 x 25 mL). The combined organic layers were washed with brine (5 x 25 mL), collected, dried over Na_2_SO_4_ and concentrated *in vacuo*. The resulting crude was purified via flash column chromatography to give **56** (48mg, 98% yield), as an off-white solid. ^1^H NMR: (300 MHz, CDCl_3_): δ 6.46 (d, *J* = 8.2 Hz, 1H), 6.80–6.84 (t, *J* = 6.3 Hz, 1H), 6.97–7.03 (m, 2H), 7.21 (d, *J* = 4.9 Hz, 1H), 7.34–7.39 (t, *J* = 7.4 Hz, 1H), 7.45–7.46 (m, 1H), 8.34–8.35 (m, 1H), 11.0 (br s, 1H); ^13^C NMR: (300 MHz, CDCl_3_): δ 104.9, 110.8, 116.2, 123.9, 124.8, 127.7, 137.4, 138.7, 143.7, 146.4, 151.3, 161.5; HRMS MS ESI *m/z* calcd for C_12_H_9_N_3_S_2_ (M+H)^+^ 260.0311, found 260.0289 (Δ 2.2 ppm).

#### *N*-(Pyridin-2-yl)-2,4'-bithiazol-2'-amine (57)

(i) To a solution of 1-(thiazol-2-yl)ethan-1-one (0.25 g, 1.97 mmol) in AcOH (5 mL), pyridinium bromide perbromide (0.82 g, 2.57 mmol) was added and allowed the mixture to stir at room temperature for 35 hours. After TLC showed completion, diluted the mixture with EtOAc (20 mL) and washed with water (3 x 10 mL). The organic layer was dried over Na_2_SO_4_ and concentrated to obtain crude 2-bromo-1-(thiazol-2-yl)ethan-1-one (310 mg, crude), which was used in next step without further purification.

(ii) To a solution of 1-(pyridin-2-yl)thiourea (0.123 g, 0.8 mmol) in DMF (7 mL), 2-bromo-1-(thiazol-2-yl)ethan-1-one (0.15 g, 0.73 mmol) and triethylamine (0.2 mL, 1.46 mmol) were added successively and heated the mixture at 70°C for 2 hours. After TLC showed completion, reaction mixture was diluted with EtOAc (20 mL) and washed with water (3 x 10 mL). The organic layer was dried over Na_2_SO_4_ and concentrated. The resulting residue was purified by column chromatography (silicagel, 100–200#) and the desired product was eluted with 40% EtOAc in hexane. Concentration of the pure fractions afforded **57** (183 mg, 96% yield), as off-white solid; ^1^H NMR: (300 MHz, CDCl_3_): δ 6.67 (d, *J* = 8.2 Hz, 1H), 6.87–6.91 (m, 1H), 7.49–7.56 (m, 2H), 7.84–7.85 (m, 1H), 8.36–8.38 (m, 1H), 9.91 (br s, 1H); ^13^C NMR: (300 MHz, CDCl_3_): δ 109.2, 110.6, 116.6, 118.9, 137.8, 143.4, 143.6, 146.7, 151.0, 161.0, 163.3; LCMS m/z (M+H) 261.02, purity 99.0%; HRMS MS ESI *m/z* calcd for C_11_H_8_N_4_S_2_ (M+H)^+^ 261.0263, found 261.0250 (Δ 1.3 ppm).

#### 4-(Pyrazin-2-yl)-*N*-(pyridin-2-yl)thiazol-2-amine (58)

To a solution of 1-(pyridin-2-yl)thiourea (0.129 g, 0.85 mmol) in DMF (7 mL), 2-bromo-1-(pyrazin-2-yl)ethan-1-one (0.20 g, 0.99 mmol) and triethylamine (0.3 mL, 2.0 mmol) were added successively and heated the mixture at 70°C for 2 hours. After TLC showed completion, reaction mixture was diluted with EtOAc (20 mL) and washed with water (3 x 10 mL). The organic layer was dried over Na_2_SO_4_ and concentrated. The resulting residue was purified by column chromatography (silicagel, 100–200#) and the desired product was eluted with 3% CH_3_OH in CH_2_Cl_2_. Concentration of the pure fractions afforded **58** (35 mg, 13.8% yield), as a greenish solid; ^1^H NMR: (400 MHz, DMSO-d_6_): δ 6.95 (d, *J* = 4.8 Hz, 1H), 7.11 (d, *J* = 8.4 Hz, 1H), 7.71–7.76 (m, 1H), 7.79 (s, 1H), 8.32–8.33 (m, 1H), 8.58 (d, *J* = 2.4 Hz, 1H), 8.65–8.66 (m, 1H), 9.16 (d, *J* = 1.6 Hz, 1H), 11.57 (s, 1H); ^13^C NMR: (300 MHz, DMSO-d_6_): 111.3, 114.5, 116.8, 117.0, 138.6, 146.8, 147.2, 152.0, 158.8, 158.9, 159.0, 160.7; LCMS m/z (M+H) 256.04, purity 98.9%; HRMS MS ESI *m/z* calcd for C_12_H_9_N_5_S (M+H)^+^ 256.06122, found 256.0634 (Δ 2.2 ppm).

#### 4-(1*H*-Pyrazol-3-yl)-*N*-(pyridin-2-yl)thiazol-2-amine (59)

(i) To a suspension of 3-methyl-1*H*-pyrazole (4.0 g, 0.048 mol) in water (100 mL), KMNO_4_ (19.24 g, 0.121 mol) was added and heated the mixture at 100°C for 4 hours. Cooled to room temperature and filtered off the solids over celite bed and concentrated the filtrate to 30 mL. The concentrated filtrate was with conc. HCl. The precipitate was collected and dried under vacuum to obtain 1*H*-pyrazole-3-carboxylic acid (2.5 g, 46% yield), as a white solid; ^1^H NMR (400 MHz, DMSO-d_6_): δ 6.71 (s, 1H), 7.75 (s, 1H), 12.61 (brs, 1H).

(ii) To a solution of 1*H*-pyrazole-3-carboxylic acid (2.5 g, 22.3 mmol) and *N*,*O*-dimethylhydroxylamine hydrochloride (2.10 g, 22.3 mmol) in CH_2_Cl_2_ (25 mL), EDCI.HCl (8.9 g, 46.6 mmol), HOBt (7.8 g, 58.2 mmol) and DIPEA (15 g, 113.7 mmol) were sequentially added and stirred at room temperature for 16 h. After completion, distilled off the solvent and the crude was purified by column chromatography on silica gel (100–200 #) using 80% EtOAc in hexane as eluent to afford *N*-methoxy-*N*-methyl-1*H*-pyrazole-3-carboxamide (3.4 g, 59% yield), as an off-white solid; ^1^H NMR (400 MHz, CDCl_3_): δ 3.39 (s, 3H), 3.78 (s, 3H), 6.83 (d, *J* = 1.2 Hz, 1H), 7.66 (d, *J* = 1.2 Hz, 1H).

(iii) To a solution of *N*-methoxy-*N*-methyl-1*H*-pyrazole-3-carboxamide (2.0 g, 12.9 mmol) and catalytic amount of DMAP in CH_2_Cl_2_ (20 mL) at 0°C, di-tert-butyl-dicarbonate (4.21 g, 19.3 mmol) was added drop wise and allowed to stir at room temperature for 2 h while monitoring by TLC. After completion, the reaction mixture was diluted with CH_2_Cl_2_ (20 mL) and washed with water (2 x 10 mL). The organic layer was concentrated under vacuum to give *tert*-butyl 3-(methoxy(methyl)carbamoyl)-1*H*-pyrazole-1-carboxylate (2.5 g, 76% yield), which was used without further purification in the next step; ^1^H NMR (400 MHz, CDCl_3_): δ 1.65 (s, 9H), 3.39 (brs, 3H), 3.81 (s, 3H), 6.82 (d, *J* = 1.2 Hz, 1H), 8.08 (d, *J* = 1.2 Hz, 1H).

(iv) To a solution of *tert*-butyl 3-(methoxy(methyl)carbamoyl)-1*H*-pyrazole-1-carboxylate (2.5 g, 9.8 mmol) in dry THF (15 mL) at -78°C under N_2_ atmosphere, methyl magnesium bromide (50 mL, 3M solution) was added drop wise and stirred at that temperature for 2 h. Then allowed the reaction to reach room temperature and continued stirring for 16 h. After TLC showed completion, the reaction was quenched with saturated ammonium chloride solution (25 mL) and extracted with EtOAc (3 x 100 mL). The combined organic layer was dried over anhydrous Na_2_SO_4_ and concentrated under reduced pressure to obtain 1-(1*H*-pyrazol-3-yl)ethan-1-one (1.0 g, 92% yield), as a semisolid; ^1^H NMR (400 MHz, CDCl_3_): δ 8.20 (brs, 1H), 7.64 (d, *J* = 1.6 Hz, 1H), 6.85 (d, *J* = 1.2 Hz, 1H), 2.59 (s, 3H).

(v) To a solution of 1-(1*H*-pyrazol-3-yl)ethan-1-one (0.80 g, 7.2 mmol) in AcOH (15 mL), pyridinium bromide perbromide (4.1 g, 14.5 mmol) was added and allowed the mixture to stir at room temperature for 24 hours. After TLC showed completion, diluted the mixture with EtOAc (30 mL) and washed with water (3 x 20 mL). The organic layer was dried over Na_2_SO_4_ and concentrated to obtain 2-bromo-1-(1*H*-pyrazol-3-yl)ethan-1-one (1.0 g, 72% yield), as a solid which was used in next step without further purification.

(vi) To a solution of 1-(pyridin-2-yl)thiourea (0.242 g, 1.58 mmol) in DMF (10 mL), 2-bromo-1-(1H-pyrazol-3-yl)ethan-1-one (0.30 g, 1.58 mmol) and triethylamine (0.44 mL, 3.16 mmol) were added successively and heated the mixture at 70°C for 2 hours. After TLC showed completion, reaction mixture was diluted with EtOAc (30 mL) and washed with water (3 x 10 mL). The organic layer was dried over Na_2_SO_4_ and concentrated. The resulting residue was purified by column chromatography (silicagel, 100–200#) and the desired product was eluted with 60% EtOAc in hexane. Concentration of the pure fractions afforded **59** (100 mg, 23% yield), as an off-white solid; ^1^H NMR: (300 MHz, CD_3_OD): δ 6.67–6.68 (m, 1H), 6.91–6.95 (m, 1H), 7.00 (d, *J* = 8.3 Hz, 1H), 7.16 (s, 1H), 7.63 (s, 1H), 7.55–7.71 (m, 1H), 8.31–8.33 (m, 1H); LCMS m/z (M+H) 244.05, purity: 94.6%; HRMS MS ESI *m/z* calcd for C_11_H_9_N_5_S (M+H)^+^ 244.0651, found 244.0631 (Δ 2.0 ppm).

#### *N*-(Pyridin-2-yl)-4-(quinolin-2-yl)thiazol-2-amine (60)

To a solution of 1-(pyridin-2-yl)thiourea (0.122 g, 0.7 mmol) in DMF (7 mL), 2-bromo-1-(quinolin-2-yl)ethan-1-one (0.20 g, 0.7 mmol) and triethylamine (0.33 mL, 2.3 mmol) were added successively and heated the mixture at 70°C for 2 hours. After TLC showed completion, the reaction mixture was diluted with EtOAc (20 mL) and washed with water (3 x 10 mL). The organic layer was dried over Na_2_SO_4_ and concentrated. The resulting residue was purified by column chromatography (silicagel, 100–200#) and the desired product was eluted with 0.5% CH_3_OH in CH_2_Cl_2_. Concentration of the pure fractions afforded **60** (45 mg, 18% yield) as a green solid; ^1^H NMR: (400 MHz, DMSO-d_6_): δ 6.95 (t, *J* = 5.6 Hz, 1H), 7.12 (d, *J* = 8.4 Hz, 1H), 7.58 (t, *J* = 7.6 Hz, 1H), 7.72–7.78 (m, 2H), 7.88 (s, 1H), 7.96–8.01 (m, 2H), 8.18 (d, *J* = 8.8 Hz, 1H), 8.34 (d, *J* = 4 Hz, 1H), 8.44 (d, *J* = 8.4 Hz, 1H), 11.54 (s, 1H); ^13^C NMR: (300 MHz, DMSO-d_6_): δ 111.3, 111.5, 111.6, 111.9, 126.6, 127.5, 128.3, 129.2, 130.3, 137.3, 137.4, 138.4, 138.5, 146.9, 148.0, 149.5, 152.2, 152.9, 160.2; LCMS m/z (M+H) 305.15, purity 98.4%; HRMS MS ESI *m/z* calcd for C_17_H_12_N_4_S (M+H)^+^ 305.0816, found 305.0838 (Δ 2.2 ppm).

#### *N*-(Pyridin-2-yl)-4-(pyrimidin-4-yl)thiazol-2-amine (61)

To a solution of 1-(pyridin-2-yl)thiourea (0.182 g, 1.19 mmol) in DMF (5 mL), 2-bromo-1-(pyrimidin-4-yl)ethan-1-one (0.20 g, 0.99 mmol) and triethylamine (0.7 mL, 5.0 mmol) were added successively and heated the mixture at 70°C for 5 hours. After TLC showed completion, the reaction mixture was diluted with EtOAc (20 mL) and washed with water (3 x 10 mL). The organic layer was dried over Na_2_SO_4_ and concentrated. The resulting residue was purified by flash column chromatography and the desired product was eluted with 5.5% CH_3_OH in CH_2_Cl_2_. Concentration of the pure fractions afforded **61** (35 mg, 13% yield), as a brown solid; ^1^H NMR: (400 MHz, DMSO-d_6_): δ 6.95–6.98 (m, 1H), 7.11 (d, *J* = 8.4 Hz, 1H), 7.76–7.71 (m, 1H), 7.90–7.93 (m, 1H), 7.96 (s, 1H), 8.33 (d, *J* = 4 Hz, 1H), 8.87 (d, *J* = 4.2 Hz, 1H), 9.16 (s, 1H), 11.54 (s, 1H); ^13^C NMR: (300 MHz, DMSO-d_6_): δ 111.3, 114.5, 116.8, 117.0, 138.6, 146.8, 147.2, 152.0, 158.8, 158.9, 159.0, 160.7; LCMS m/z (M+H) 256.04, purity 95.0%; HRMS MS ESI *m/z* calcd for C_12_H_9_N_5_S (M+H)^+^ 256.0651, found 256.0625 (Δ 2.6 ppm).

#### 1- [2-(Pyridin-2-yl)-thiazol-4-yl]ethan-1-one (62)

(i) To a solution of 2-(pyridin-2-yl)thiazole-4-carboxylic acid (250 mg, 1.21 mmol) and *N*,*O*-dimethylhydroxylamine hydrochloride (178 mg, 1.82 mmol) in CH_2_Cl_2_ (10 mL), EDCI.HCl (341 mg, 1.82 mmol), HOBt (245 mg, 1.82 mmol) and DIPEA (0.55 mL, 3.02 mmol) were sequentially added and stirred at room temperature for 16 h. After completion, the reaction was diluted with water (10 mL) and extracted with CH_2_Cl_2_ (2 x 15 mL). The combined organic layer was dried over Na_2_SO_4_ and concentrated under reduced pressure. The crude was further purified by column chromatography on silica gel (100–200 #) using 30% EtOAc in hexane as eluent to afford *N*-methoxy-*N*-methyl-2-(pyridin-2-yl)thiazole-4-carboxamide (130 mg, 43% yield), as an off-white solid; ^1^H NMR (400 MHz, CDCl_3_): δ 8.62 (d, *J* = 4.4 Hz, 1H), 8.23 (d, *J* = 8.0 Hz, 1H), 8.12 (s, 1H), 7.81(t, *J* = 7.6 Hz, 1H), 7.35 (t, *J* = 7.6 Hz, 3H), 3.86 (s, 3H), 3.48 (s, 3H).

(ii) To a solution of N-methoxy-N-methyl-2-(pyridin-2-yl)thiazole-4-carboxamide (50 mg, 0.2 mmol) in dry THF (5 mL) at -78°C under N_2_ atmosphere, methyl magnesium bromide (3M solution, 1.0 mL) was added drop wise and stirred at -78°C for 4 hours. After TLC showed completion, quenched the reaction with saturated ammonium chloride solution (5 mL) and extracted with EtOAc (3 x 15 mL). The combined organic layer was dried over anhydrous Na_2_SO_4_ and concentrated under reduced pressure. The crude was further purified by column chromatography on silica gel (100–200 #) using 30% EtOAc in hexane as eluent to obtain **62** (20 mg, 49% yield), as brown solid; ^1^H NMR: (300 MHz, CD_3_OD): δ 2.25 (s, 3H), 7.32–7.36 (m, 1H), 7.74 (s, 1H), 7.86–7.91 (m, 1H), 8.07 (d, *J* = 7.9 Hz, 1H), 8.55–8.56 (m, 1H); ^13^C NMR: (300 MHz, CD_3_OD): δ 21.1, 111.3, 121.0, 122.6, 137.4, 148.6; LCMS m/z (M+H) 205.02, purity: 99.4%; HRMS MS ESI *m/z* calcd for C_10_H_8_N_2_OS (M+H)^+^ 205.043, found 205.0425 (Δ 0.5 ppm).

#### *N*-(4-Acetylthiazol-2-yl)adamantane-1-carboxamide (63)

To a solution of 2-(adamantane-1-carboxamido)-*N*-methoxy-*N*-methylthiazole-4-carboxamide (250 mg, 0.7 mmol) in dry THF (10 mL) at -78°C under N_2_ atmosphere, methyl magnesium bromide (3 M solution, 1.05 mmol) was added drop wise and stirred at -78°C for 2 hours. After TLC showed completion, quenched the reaction with saturated ammonium chloride solution (10 mL) and extracted with EtOAc (3 x 25 mL). The combined organic layer was dried over anhydrous Na_2_SO_4_ and concentrated under reduced pressure. The crude was further purified by column chromatography on silica gel (100–200 #) using 10% EtOAc in hexane as eluent to obtain **63** (60 mg, 28% yield), as off-white solid; ^1^H NMR: (300 MHz, CDCl_3_): δ 1.74–1.85 (m, 6H), 1.99 (s, 6H), 2.45 (s, 3H), 2.60 (s, 3H), 7.79 (s, 1H), 8.94 (br s, 1H); ^13^C NMR: (300 MHz, CDCl_3_): δ 27.1, 27.8, 36.2, 38.8, 121.4; LCMS m/z (M+H) 305.15, purity: 99.9%. HRMS MS ESI *m/z* calcd for C_16_H_20_N_2_O_2_S (M+H)^+^ 305.1318, found 305.1305 (Δ 1.3 ppm).

#### *N*- [5- [4-(1,1-Dimethylethyl)phenyl]-1,3,4-thiadiazol-2-yl]-2-pyridinecarboxamide (64)

To 5-(2-pyridinyl)-1,3,4-thiadiazol-2-amine (18 mg, 0.1 mmol) dissolved in ethanol (1 mL) was added 4-(1,1-dimethylethyl)benzoyl chloride (0.028 mL, 0.1 mmol). The mixture was stirred at room temperature and the reaction was monitored via LC/MS. As the reaction progressed, precipitate formed. After 1 h, the precipitate was collected via vacuum filtration and washed with H_2_O. The resulting solid was recrystallized from ethanol to give 64 (13 mg, 38% yield), as a white solid; ^1^H NMR: (300 MHz, CDCl_3_): δ 1.41 (s, 9H), 7.37–7.41 (m, 2H), 7.60 (d, *J* = 8.3 Hz, 2H), 7.80–7.85 (m, 1H), 8.20–8.28 (m, 3H), 8.69–8.71 (m, 1H), 12.12 (br s, 1H); ^13^C NMR: (300 MHz, CDCl_3_): δ 31.1, 35.2, 45.5, 73.0, 74.8, 100.8, 104.1, 108.0, 120.2, 124.8, 125.8, 128.5, 136.7, 149.8; HRMS MS ESI *m/z* calcd for C_18_H_18_N_4_OS (M+H)^+^ 339.1274, found 339.1256 (Δ 1.8 ppm).

#### *N*-(2,6-Dimethylphenyl)-5-(pyridin-2-yl)-1,3,4-thiadiazol-2-amine (65)

To a solution of picolinohydrazide (500 mg, 3.65 mmol) in ethanol (10 mL), 2-isothiocyanato-1,3-dimethylbenzene (595 mg, 3.65 mmol) was added and the mixture was refluxed for 15 h. After TLC showed completion, distilled off the solvent and the residue was washed with diethyl ether to obtain N-(2,6-dimethylphenyl)-2-picolinoylhydrazine-1-carbothioamide as off-white solid (1.0 g, 91%). MS m/z (M+H) 301.23

A mixture of N-(2,6-dimethylphenyl)-2-picolinoylhydrazinecarbothioamide (200 mg, 0.66 mmol) and conc. H_2_SO_4_ (1 mL) was stirred at room temperature for 8 h while monitoring by TLC analysis (CH_3_OH:CH_2_Cl_2_ = 1:19). The reaction mixture was poured into ice cooled water (10 mL) and extracted with EtOAc (3 x 20 mL). The combined organic layer was dried over anhydrous Na_2_SO_4_ and concentrated under reduced pressure. The crude was further purified by column chromatography on silica gel (100–200 mesh) using 1% CH_3_OH in CH_2_Cl_2_ as eluent to afford **65** (30 mg, 16%), as a white solid; ^1^H NMR: (400 MHz, DMSO-d_6_): δ 2.22 (s, 6H), 7.17 (s, 3H), 7.40–7.44 (m, 1H), 7.90–7.94 (m, 1H), 8.05 (d, *J* = 8 Hz, 1H), 8.54–8.55 (m, 1H), 9.67 (s, 1H); ^13^C NMR: (300 MHz, CDCl_3_): δ 18.1, 119.7, 124.0, 128.1, 129.0, 136.4, 136.6, 136.7, 138.3, 149.22, 149.29, 149.9, 173.4; LCMS m/z (M+H) 283.08, purity 99.4%; HRMS MS ESI *m/z* calcd for C_15_H_14_N_4_S (M+H)^+^ 283.1012, found 283.1005 (Δ 0.7 ppm).

#### *N*-(2,6-Dimethylphenyl)-5-(pyridin-2-yl)-1,3,4-oxadiazol-2-amine (66)

To a solution of picolinohydrazide (500 mg, 3.65 mmol) in ethanol (10 mL), 2-isothiocyanato-1,3-dimethylbenzene (595 mg, 3.65 mmol) was added and the mixture was refluxed for 15 h. After TLC showed completion, distilled off the solvent and the residue was washed with diethyl ether to obtain N-(2,6-dimethylphenyl)-2-picolinoylhydrazine-1-carbothioamide as off-white solid (1.0 g, 91%). MS m/z (M+H) 301.23

A mixture of N-(2,6-dimethylphenyl)-2-picolinoylhydrazinecarbothioamide (200 mg, 0.66 mmol) and EDC.HCl (511 mg, 2.66 mmol) in CH_2_Cl_2_ (10 mL) was stirred at room temperature for 15 h while monitoring by TLC analysis (CH_3_OH:CH_2_Cl_2_ = 1:19). The reaction mixture was poured into ice cooled water (10 mL) and extracted with CH_2_Cl_2_ (3 x 20 mL). The combined organic layer was dried over anhydrous Na_2_SO_4_ and concentrated under reduced pressure. The crude was further purified by column chromatography on silica gel (100–200 mesh) using 1% CH_3_OH in CH_2_Cl_2_ as eluent to afford **66** (70 mg, 39%), as a white solid; ^1^H NMR: (400 MHz, DMSO-d_6_): δ 2.21 (s, 6H), 7.14 (s, 3H), 7.50–7.53 (m, 1H), 7.94–8.02 (m, 2H), 8.66 (d, *J* = 4.4 Hz, 1H), 9.65 (s, 1H); ^13^C NMR: (300 MHz, CDCl_3_): δ 16.8, 121.7, 125.3, 127.3, 128.2, 135.7, 137.7, 149.5; LCMS m/z (M+H) 267.11, purity 99.1%; HRMS MS ESI *m/z* calcd for C_15_H_14_N_4_O (M+H)^+^ 267.124, found 267.1224 (Δ 1.6 ppm).

#### *N*-(2,6-Dimethylphenyl)-6-(pyridin-2-yl)pyrimidin-4-amine(67)

A mixture of 6-chloro-N-(2,6-dimethylphenyl)pyrimidin-4-amine **12** (200 mg, 0.85 mmol), 2-(1,1,1-tributylstannyl)pyridine (631 mg, 1.7 mmol) in dry DMF (8 mL) in a seal tube was degassed for 20 minutes and Pd(PPh_3_)_4_ (148 mg, 0.12 mmol) was added. The contents in the sealed tube were heated at 100°C for 16 hours. After TLC showed completion, diluted with water (15 mL) and extracted with EtOAc (3 x 30 mL). The organic layer was dried over Na_2_SO_4_ and concentrated. The resulting residue was purified by column chromatography followed by prep-HPLC to obtain **67** (50 mg, 21% yield), as a white solid; ^1^H NMR: (400 MHz, DMSO-d_6_): δ 2.14 (s, 6H), 6.73–6.77 (br s, 1H), 7.11–7.22 (m, 3H), 7.50 (d, *J* = 16.4 Hz, 1H), 7.94 (s, 1H), 8.33 (d, *J* = 8 Hz, 1H), 8.45–8.71 (m, 2H), 9.09 (s, 1H); ^13^C NMR: (300 MHz, CDCl_3_): δ 18.4, 121.6, 124.8, 127.7, 128.7, 128.9, 136.6, 136.9, 149.2, 158.5; LCMS m/z (M+H) 277.17, purity: 99.5%; HRMS MS ESI *m/z* calcd for C_17_H_16_N_4_ (M+H)^+^ 277.1448, found 277.1438 (Δ 1.0 ppm).

#### 4-*tert*-Butyl-*N*- [4-(pyridin-2-yl)pyrimidin-2-yl]benzamide (68)

To a stirred solution of MsCl (93 mg, 0.81 mmol) in DMA (4 mL) at 0°C, under N_2_ atmosphere, a solution of 4-(*tert*-butyl)benzoic acid (105 mg, 0.58 mmol) and 2,6-lutidine (0.18 mL, 1.6 mmol) in DMA (2 mL) was added and stirred for 15 minutes. To this mixture was added a solution of 4-(pyridin-2-yl)pyrimidin-2-amine (100 mg, 0.58 mmol) in DMA (2 mL). The reaction mixture was heated at 50°C for 3 hours while monitoring by TLC. After completion, the reaction mixture was diluted with water (15 mL) and extracted with EtOAc (3 x25 mL). The organic layer was dried over Na_2_SO_4_ and concentrated. The resulting crude was purified by column chromatography on silica gel (100–200 mesh) using 1% CH_3_OH in CH_2_Cl_2_ as eluent to obtain **68** (33 mg, 17% yield), as a brown solid; ^1^H NMR: (300 MHz, CDCl_3_): δ 1.39 (s, 9H), 7.42–7.46 (m, 1H), 7.54 (d, *J* = 8.3 Hz, 2H), 7.86–7.89 (m, 1H), 7.92 (d, *J* = 8.4 Hz, 2H), 8.11 (d, *J* = 5.1 Hz, 1H), 8.51 (d, *J* = 7.9 Hz, 1H), 8.70 (s, 1H), 8.74–8.76 (m, 1H), 8.82 (d, *J* = 5.2 Hz, 1H); ^13^C NMR: (300 MHz, CDCl_3_): δ 31.1, 112.9, 122.1, 125.6, 125.8, 127.3, 137.2, 148.5, 149.5, 159.6; LCMS m/z (M-H) 333.21, purity: 96.3%; HRMS MS ESI *m/z* calcd for C_20_H_20_N_4_O (M+H)^+^ 333.1671, found 333.1685 (Δ 1.4 ppm).

#### *N*- [1-(Pyridin-2-yl)-1H-pyrazol-3-yl]adamantane-1-carboxamide (69)

To 1-(2-Pyridyl)-1H-pyrazole-3-amine (**15**, 32 mg, 0.2 mmol) dissolved in CH_2_Cl_2_ (1 mL) was added tricyclo [3.3.1.1^3,​7^]decane-1-carbonyl chloride (48 mg, 0.24 mmol). Upon addition, precipitate formed. The mixture was stirred at room temperature and the reaction was monitored via LC/MS. After 1 h, the reaction was diluted in 5% K_2_CO_3_ in water (2 mL). The aqueous layer was extracted with EtOAc (3 x 25 mL). The organic layers were combined, dried over Na_2_SO_4_ and concentrated *in vacuo*. The resulting crude was purified via flash column chromatography to give **69** (41 mg, 64% yield), as a white solid; ^1^H NMR: (300 MHz, CDCl_3_): δ 1.77–1.83 (m, 6H), 2.00 (s, 6H), 2.12 (s, 3H), 7.03 (s, 1H), 7.16–7.18 (m, 1H), 7.74–7.82 (m, 2H), 8.09 (s, 1H), 8.40–8.41 (m, 1H), 8.44 (br s, 1H); ^13^C NMR: (300 MHz, CDCl_3_): δ 28.0, 36.4, 39.1, 100.6, 111.4, 120.9, 127.8, 138.6, 148.1; HRMS MS ESI *m/z* calcd for C_19_H_22_N_4_O (M+H)^+^ 323.1827, found 323.1853 (Δ 2.6 ppm).

#### 3-Cyclopentyl-1- [1-(pyridin-2-yl)-*1H*-pyrazol-3-yl]urea (70)

To 1-(2-Pyridyl)-1H-pyrazole-3-amine (**15**, 32 mg, 0.2 mmol) dissolved in CH_2_Cl_2_ (1 mL) was added cyclopentane​carbonyl chloride (0.04 mL, 0.24 mmol). Upon addition, precipitate formed. The mixture was stirred at room temperature and the reaction was monitored via LC/MS. After 1 h, the reaction was diluted in 5% K_2_CO_3_ in water (2 mL). The aqueous layer was extracted with EtOAc (3 x 25 mL). The organic layers were combined, dried over Na_2_SO_4_ and concentrated *in vacuo*. The resulting crude was purified via flash column chromatography to give **70** (35 mg, 36% yield), as a white solid; ^1^H NMR: (300 MHz, CDCl_3_): δ 1.60–1.80 (m, 7H), 2.07–2.09 (m, 2H), 4.25–4.31 (m, 1H), 6.11 (s, 1H), 7.12–7.16 (m, 1H), 7.60 (d, *J* = 8.1 Hz, 1H), 7.78–7.83 (t, *J* = 7.4 Hz, 1H), 7.97 (br s, 1H), 8.38–8.43 (m, 2H), 8.59 (s, 1H); ^13^C NMR: (300 MHz, CDCl_3_): 23.5, 33.5, 51.9, 98.0, 110.8, 120.5, 127.8, 138.6, 148.1, 150.8, 151.2, 155.1; HRMS MS ESI *m/z* calcd for C_14_H_17_N_5_O (M+H)^+^ 272.1467, found 272.1493 (Δ 2.6 ppm).

## Abbreviations

AT: aminothiazole
MIC: minimum inhibitory concentration
TC50: half-maximum inhibitory concentration
MBC: minimum bactericidal concentration
SI: selectivity index

## References

[1] World Health Organization Global tuberculosis report 2014.

[2] Mitchison D, Davies G. The chemotherapy of tuberculosis: past, present and future. Int J Tuberc Lung Dis. 2012;16(6):724–32. 10.5588/ijtld.12.0083 22613684 PMC3736084

[3] Pethe K, Sequeira PC, Agarwalla S, Rhee K, Kuhen K, Phong WY, et al A chemical genetic screen in *Mycobacterium tuberculosis* identifies carbon-source-dependent growth inhibitors devoid of *in vivo* efficacy. Nat Commun. 2010;1(5):1–8. 20975714 10.1038/ncomms1060 PMC3220188

[4] Ballell L, Bates RH, Young RJ, Alvarez-Gomez D, Alvarez-Ruiz E, Barroso V, et al Fueling open-source drug discovery: 177 small-molecule leads against tuberculosis. ChemMedChem. 2013;8(2):313–21. 10.1002/cmdc.201200428 23307663 PMC3743164

[5] Ananthan S, Faaleolea ER, Goldman RC, Hobrath JV, Kwong CD, Laughon BE, et al High-throughput screening for inhibitors of *Mycobacterium tuberculosis* H37Rv. Tuberculosis (Edinb). 2009;89(5):334–53. 19758845 10.1016/j.tube.2009.05.008 PMC3255569

[6] Luger P, Daneck K, Engel W, Trummlitz G, Wagner K. Structure and physicochemical properties of meloxicam, a new NSAID. Eur J Pharm Sci. 1996;4(3):175–87.

[7] Das J, Chen P, Norris D, Padmanabha R, Lin J, Moquin RV, et al 2-Aminothiazole as a novel kinase inhibitor template. structure−activity relationship studies toward the discovery of N-(2-Chloro-6-methylphenyl)-2-[[6-[4-(2-hydroxyethyl)-1- piperazinyl)]-2-methyl-4-pyrimidinyl]amino)]-1,3-thiazole-5-carboxamide (Dasatinib, BMS-354825) as a potent pan-src kinase inhibitor. J Med Chem. 2006;49(23):6819–32. 17154512 10.1021/jm060727j

[8] Inamoto Y, Chiba T, Kamimura T, Takaya T. FK 482, a new orally active cephalosporin synthesis and biological properties. J Antibiot (Tokyo). 1988;41(6):828–30. 3255303 10.7164/antibiotics.41.828

[9] Prasada Rao KVV, Dandala R, Sivakumaran M, Rani A, Naidu A. Novel compounds for the synthesis of cefdinir. J Heterocycl Chem. 2007;44(2):309–14.

[10] Meissner A, Boshoff HI, Vasan M, Duckworth BP, Barry CE, 3rd., Aldrich CC. Structure-activity relationships of 2-aminothiazoles effective against *Mycobacterium tuberculosis*. Bioorg Med Chem. 2013;21(21):6385–97. 10.1016/j.bmc.2013.08.048 24075144 PMC3816974

[11] Mjambili F, Njoroge M, Naran K, De Kock C, Smith PJ, Mizrahi V, et al Synthesis and biological evaluation of 2-aminothiazole derivatives as antimycobacterial and antiplasmodial agents. Bioorg Med Chem Lett. 2014;24(2):560–4. 10.1016/j.bmcl.2013.12.022 24373723

[12] Hantzsch A. Über die oxy-thiazole oder thiazolone. Ber dtsch Chem Ges A/B. 1927;60(2537–2545).

[13] Egan RS, Tadanier J, Garmaise DL, Gaunce AP. Intermediates in the Hantzsch thiazole synthesis. J Org Chem. 1968;33(12):4422–6.

[14] Taurins A, Blaga A. Synthesis of pyridyl- and quinolyl-substituted 2-aminothiazoles. J Heterocycl Chem. 1970;7(5):1137–41.

[15] Ollinger J, Bailey MA, Moraski GC, Casey A, Florio S, Alling T, et al A dual read-out assay to evaluate the potency of compounds active against *Mycobacterium tuberculosis*. PLoS One. 2013;8(4):e60531 10.1371/journal.pone.0060531 23593234 PMC3617142

[16] Sirgel FA, Wiid IJ, van Helden PD. Measuring minimum inhibitory concentrations in mycobacteria In: Parish T, Brown AC, editors. Mycobacteria Protocols. 465 Totowa, NJ: Humana Press; 2009 p. 173–86. 10.1007/978-1-59745-207-6_11 20560078

[17] Garcia RJL, Pedregal C, Rodriguez JH. Synthesis and conformational analysis of some oxisuran metabolites and their O-methyl derivatives. Tetrahedron. 1987;43(19):4407–16.

[18] Frigola J, Colombo A, Pares J, Martinez L, Sagarra R, Roser R. Synthesis, structure and inhibitory effects on cyclooxygenase, lipoxygenase, thromboxane synthetase and platelet aggregation of 3-amino-4,5-dihydro-1H-pyrazole derivatives. Eur J Med Chem. 1989;24(4):435–45.

[19] Aicher B, Kelter A-R, Coulter TS, Taylor S, Davenport AJ, Hesterkamp T, et al., inventors; Evotec A.-G., assignee. Preparation of piperazine(thio)​carboxamides as inhibitors of hematopoietic prostaglandin D synthase. Germany 2010.

